# Correction: Double-blind randomized N-of-1 trial of transcranial alternating current stimulation for mal de débarquement syndrome

**DOI:** 10.1371/journal.pone.0315783

**Published:** 2024-12-10

**Authors:** The PLOS ONE Staff

## Notice of Republication

This article was republished on 3 January 2023, to correct for errors in the Data Availability statement. The publisher apologizes for the errors. Please download this article again to view the correct version. The originally published, uncorrected article and the republished, corrected article are provided here for reference.

## Supporting information

S1 File Originally published, uncorrected article. (PDF)

S2 File Republished, corrected article. (PDF)

## References

[1] Cha Y-H, Gleghorn D, Doudican BC (2022) Double-blind randomized N-of-1 trial of transcranial alternating current stimulation for mal de débarquement syndrome. PLoS ONE 17(2): e0263558. 10.1371/journal.pone.0263558 35120184 PMC8815977

