# Supplementary figures and images for: Double-blind randomized N-of-1 trial of transcranial alternating current stimulation for mal de débarquement syndrome
Source: PLoS One. 2022 Feb 4;17(2):e0263558. doi: 10.1371/journal.pone.0263558 (PMC8815977; doi:10.1371/journal.pone.0263558)

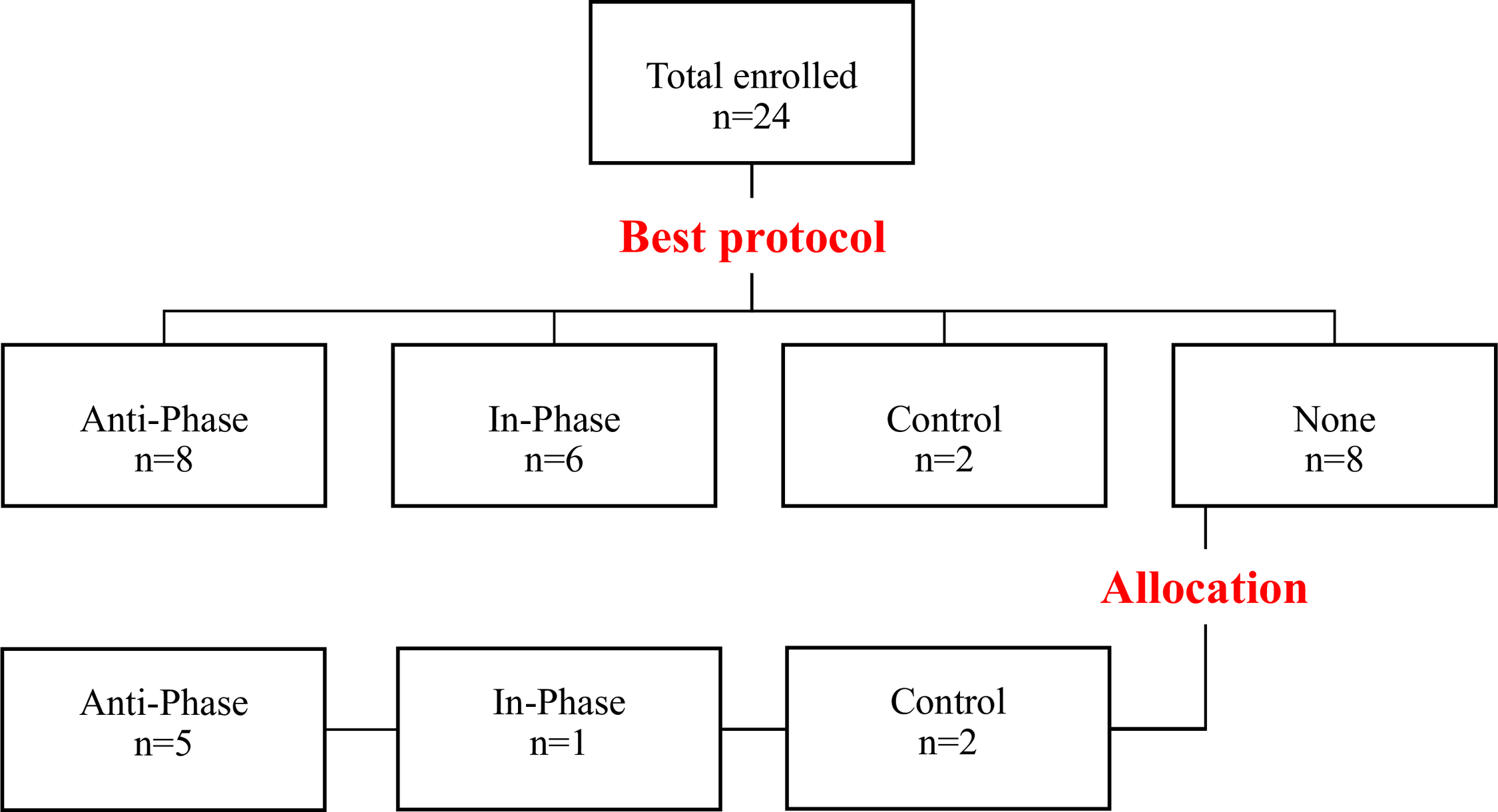

Supplement: S1 Fig — (TIF) [file pone.0263558.s002.tif]

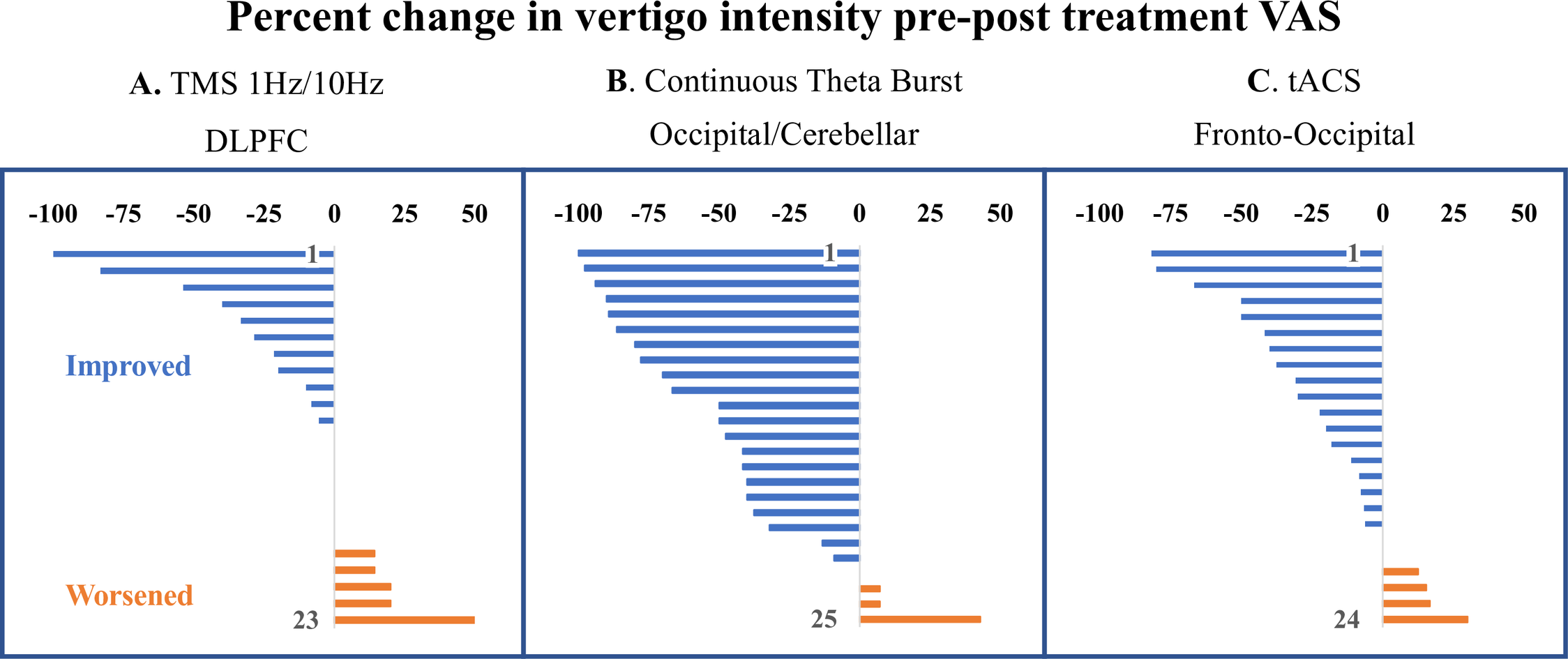

Supplement: S2 Fig — A. Transcranial magnetic stimulation (TMS) at 1Hz and 10Hz over dorsolateral prefrontal cortex (DLPFC), B. Continuous theta burst stimulation over occipital cortex and cerebellar vermis, C. Transcranial alternating current stimulation (tACS) over fronto-occipital cortex. (TIF) [file pone.0263558.s003.tif]

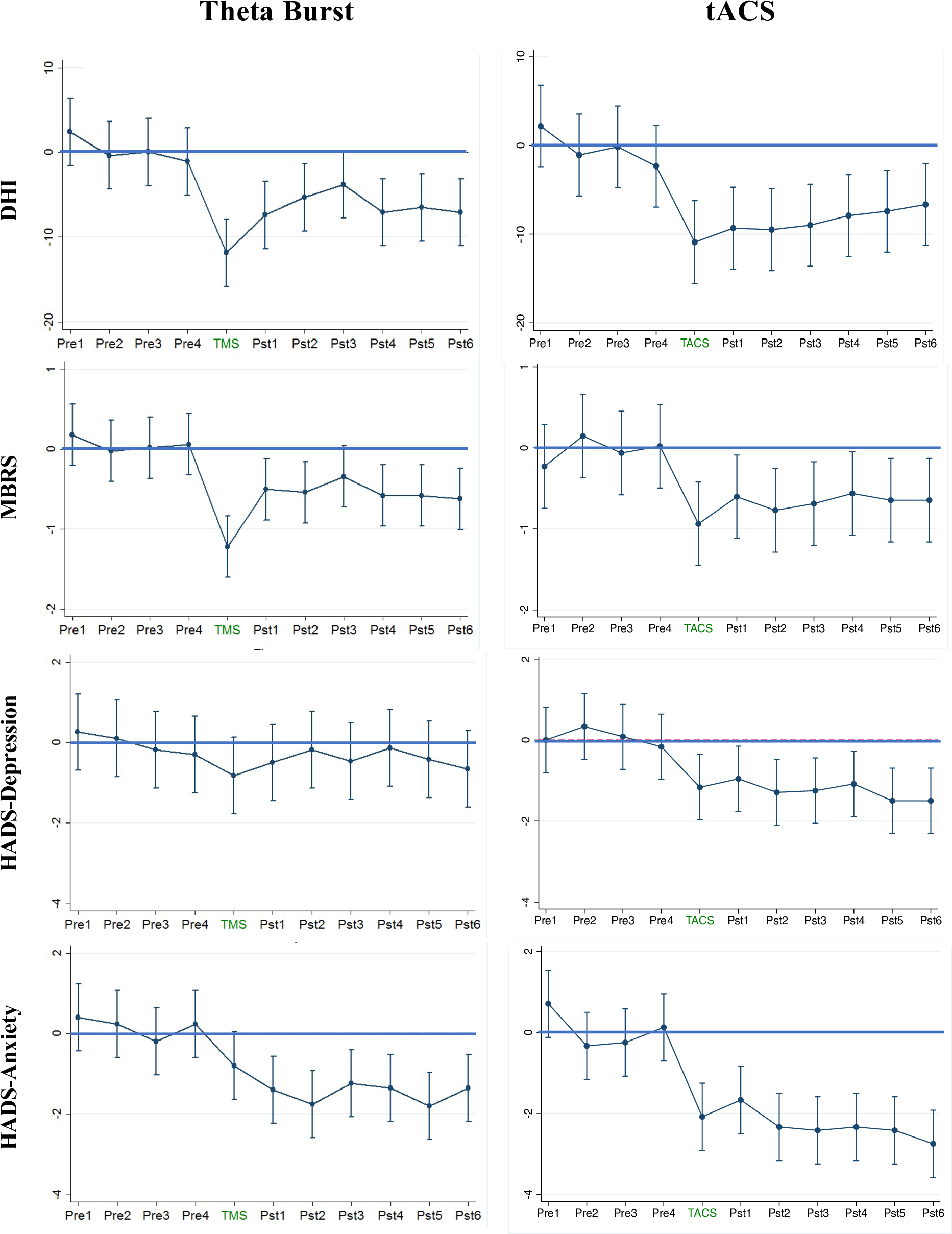

Supplement: S3 Fig — Mean score changes from baseline and 95% confidence interval for A. Dizziness Handicap Inventory, B. MdDS Balance Rating Scale, C. Hospital Anxiety and Depression Scale-Depression subscore, and D. Hospital Anxiety and Depression Scale-Anxiety subscore. (TIF) [file pone.0263558.s004.tif]
