# Supplementary material for: Double-blind randomized N-of-1 trial of transcranial alternating current stimulation for mal de débarquement syndrome
Source: PLoS One. 2022 Feb 4;17(2):e0263558. doi: 10.1371/journal.pone.0263558 (PMC8815977; doi:10.1371/journal.pone.0263558)
Supplement: S1 File — (DOCX) [file pone.0263558.s005.docx]

**Title: Trans**cranial Electrical Stimulation for Mal de Debarquement Syndrome

**Principal Investigator:**

Yoon-Hee Cha, MD Assistant Professor, Laureate Institute for Brain Research (LIBR), Adjunct Assistant Professor, UCLA School of Medicine

**Sponsor:** Laureate Institute for Brain Research, Inc.

**Abstract**

*Mal de debarquement syndrome* (MdDS) is a balance disorder in which individuals develop a persistent internal sense of movement after a prolonged period of passive motion exposure. This is classically described as "land-sickness" occurring after boat rides. Patients describe a hallucination of movement such as "rocking" and "bobbing" even though they are not physically moving. This disorder is thought to be due to a primary problem of brain adaptation as testing of inner ear function and structural brain imaging is always normal. However, it is unknown which parts of the brain control this adaptation. Most cases of *mal de debarquement* only last a few hours, but there are many people who experience the symptoms for months or years, leading to significant morbidity. The term MdDS is generally reserved for patients who experience symptoms for at least one month.

Our preliminary data using transcranial magnetic stimulation (TMS) has shown that TMS can be very effective in some individuals with MdDS. TMS is a form of neuromodulation using a fluctuating magnetic field placed over the brain area of interest to modulate brain activity. Although TMS has the advantage of giving very focal stimulation, it is expensive both in upfront costs and set-up time. It also requires that subjects come to the research site every day. An alternative to TMS is transcranial electrical stimulation (TES). There are many forms of TES: those that use direct current, alternating current, or random noise current. TES can also be given via a peripheral nerve such as through the vestibular nerve, a process referred to as galvanic vestibular stimulation (GVS). GVS can be used as a “bottom-up” method to alter cerebral functioning. A TES device that can give either direct or alternating current has FDA approval and is currently marketed to the public (Fisher Wallace®). They all, however, use a very low level of current (2mA or less) administered on the head through two electrodes. TES studies in disorders such as depression, tinnitus, chronic pain, and stroke rehabilitation have shown that many of the physiological changes that are achieved with TMS can also be achieved with TES. The advantage of TES is that it can be administered by the subjects on themselves, is significantly less expensive and thus can be used for a longer treatment period.

Given that MdDS is a rare disorder with symptoms that are worsened by travel, we aim to explore the use of TES for this disorder in order to provide subjects an option for treatment that is coupled with minimized risk of exacerbating their symptoms. Our goals are to determine whether TES can extend the benefit of TMS, whether it can work as a standalone therapy, and to determine how TES and GVS can alter cerebral functional connectivity.

**A. Specific Aims**

The goal of this study is to determine whether external neuromodulation using TES or GVS can reduce the perception of self-motion that is experienced by patients with MdDS. We will also compare how these interventions affect brain connectivity in healthy controls via EEG and fMRI. We will determine the optimal treatment duration and stimulation parameters. Our goal is to make the study as flexible as possible for the participants in order to maintain compliance and encourage enrollment.

**B. Background and Significance**

Mal de debarquement syndrome (MdDS), the term for persistent feelings of motion occurring after exposure to passive motion, was reported over 200 years ago, but there is still no explanation for why some people experience such persistent feelings of rocking and swaying after being exposed to prolonged but relatively minor amplitudes of motion. The most common trigger for MdDS is sea travel with the quintessential patient being a middle-aged woman who develops sensations of rocking and swaying after disembarking from a cruise. Although brief periods of self-motion perception are common, some people experience the chronic rocking sensation for months or years with very few options for symptom relief. These patients can become totally disabled, often stopping work, developing strained relationships, and experiencing severely reduced quality of life. The probability of symptoms spontaneously stopping decreases dramatically with longer durations of symptoms. Thus, patients who have had MdDS for more than a year have a limited chance for recovery and almost no therapeutic recourse if one or two medication trials have failed them.

MdDS is unique among balance disorders in that re-exposure to passive motion decreases the internal motion perception rather than increasing feelings of motion sickness. Vestibular function testing, structural brain, and inner ear imaging are non-diagnostic. Various theories have been proposed to explain MdDS ranging from abnormal weighting of somatosensory inputs, to de-afferentation of vestibular signals, to persistent internal models of external stimuli. However, one major barrier to progress in understanding the basis of self-motion perception has been the limitation of diagnostic and therapeutic tools.

More options are needed for treating patients with MdDS because they do not respond to the typical therapies given for balance disorders. Vestibular therapy and vestibular suppressants are not helpful and sometimes worsen symptoms. Benzodiazepines provide palliation only, and limited data on the use of selective serotonin reuptake inhibitors shows that, though they are helpful in some patients, are not effective in many others.

Our preliminary data shows that external brain neuromodulation with TMS can be effective in MdDS and that one form of TES tested, called transcranial direct current stimulation (tDCS), can also be effective (Figure 1). tDCS is a specific form of CES which uses directionally applied current to modulate brain function. tDCS employs polarity differences of direct current to stimulate or inhibit underlying brain areas. Current flows from the anode to the cathode creating a net negative charge of the extracellular fluid under the anode, and more positive charge under the cathode. This lowers the membrane potential difference under the anode, bringing cells closer to depolarization and increasing activity. The opposite effect creates hyperpolarization under the cathode, leading to suppressed activity. Despite using very low-level current in the 1-2mA range, studies employing tDCS have shown positive therapeutic effects in disorders that range from depression, to tinnitus, stroke, and others.

Transcranial alternating current stimulation (tACS) is similar to tDCS except that the current continuously changes direction, typically at a frequency of 10-500Hz. Since there is no polarity difference, tACS studies generally place the electrodes symmetrically on the head. Transcranial random noise stimulation (tRNS) uses similar current and electrodes but give a current profile that continuously changes and has no specific pattern. GVS can be given either with directional current or with sinusoidal current in the form of tACS. There is already FDA approval for tDCS and tACS through a company called Fisher Wallace®. This device has approval for the treatment of depression, anxiety, and insomnia. Although this company’s device is not suitable for this study because it does not have any automatic shut-off features for safety, there is recognition by the FDA of the safety and efficacy of TES. It should be noted that there is great public interest in the use of TES and several companies have appeared in the last one-year providing advice to the lay public on how to create these devices by themselves at home with simple Do-It-Yourself kits.

The set up for the stimulation sessions involves placing the two electrodes over target brain areas or over the mastoid processes. The electrodes consist of saline soaked sponges housing carbonized rubber sponges that deliver the current. The electrodes are generally held in place with a headband. Stimulation sessions for human studies range from 10-30minutes, at a current setting of 1-2mA. Given the surface area of sponges used in TES studies, the current densities used range from 0.01-0.08mA/cm2, which are about 0.04-0.3% of the lower limit of what has been determined to potentially cause brain tissue injury.

Safety studies of TES show that the most common adverse effects are issues such as itching, paresthesias, and headache, rather than serious potential side effects like seizures in repetitive transcranial stimulation (rTMS), the most popular form of neuromodulation. The rare but serious main concern with TES is the possibility of skin burns that can occur when the electrodes are not sufficiently wet, when they are applied over broken skin, or when the salt content of the conductive solution is not high enough (as when using water instead of saline). Guidelines for proper application for TES have been published, that reference up to 30 sequentlal sessions of tDCS being performed safely. Given these safety concerns, however, our study will be using a stimulation device that automatically shuts off when stimulation exceeds a set resistance.

The hypothesis of this study is that external neuromodulation with TES over target brain areas or the vestibular nerve can reduce the perception of motion or alter functional connectivity. Questions to be answered during the study are what factors are important in treatment response such as: duration of illness, concurrent medications, specific electrode placement, duration of treatment, etc. By making a more flexible treatment regimen be available to participants, we can determine the optimal course of treatment for this disorder.

**D. Research Design and Methods**

**Screening:** All potential subjects will be screened by the PI or trained study staff to determine whether they meet any exclusion criteria (see attached screening script) such as any major medical or psychiatric illness that would preclude the use of TES. All screening will occur prior to participation and will be either in person or on the phone.

**Consent:** The purpose, content, duration, and expected risks and benefits of the study will be reviewed with each participant by the PI or trained study staff. If they pass the screening interview, written consent will also be obtained before subjects can participate in any portion of the study. The actual consent form will either be mailed or emailed to the potential participant, depending on their preference. Subjects will be encouraged to be open with the investigators about any questions or concerns they have either before, during, or after the study. For studies in which a portable stimulator is sent home, e.g. the TCT or Pulvinar stimulators, a “study buddy” who can assist with the application of the cap will also be consented. We have been able to show in a study of 23 subjects that they can perform the stimulations safely on themselves without skin burns. They are also responsible enough to return the stimulators to us (Cha et. al. Brain Stimulation, 2016). Note, stimulators will only be sent home with the MdDS subjects. Healthy controls will only be tested on-site.

**Enrollment:** All subjects will be encouraged to be enrolled by coming to the research site and meet with the PI and study staff directly. However, if they are unable to travel to the study site because of the severity of their symptoms or because of financial constraints, they may be enrolled through a webcam interview. This is possible because TES is very straightforward to apply and people with MdDS are very healthy otherwise. They almost always have normal neurological exams and any abnormalities are what are general expected in the general population (eg. an occasional neuropathy). Subjects will be asked to provide records of any testing that they have had, such as MRI scans of the brain, hearing tests, or inner ear function testing, or any prior evaluations that had led to their diagnosis.

If the subject is not enrolled in person, they will be sent the device ahead of time. The instructions for applying the TES electrodes can be taught over a webcam connection. There are also a series of instructional videos that the subjects can refer to: http://www.trans-cranial.com/manuals.

All subjects will complete some or all of the following questionnaires, which will be tailored to their specific situation (eg. subjects without a history of migraine would not be given the MIDAS): ABC Scale, Beck Depression Short Form, Cognitive Assessment Questionnaire, Dizziness Handicap Inventory, Edinburgh Handedness Scale, Functional Activities Questionnaire, Hospital Depression Anxiety Scale, Mal de Debarquement Rating Scale, Multidimensional Fatigue Scale, MIDAS Migraine Disability Scale (if applicable), Motion Sensitivity Questionnaire, MSSQ, Memory Questionnaire, SF-36 (quality of life scale), Tinnitus Handicap Inventory (if applicable), an Empathy Scale a Visual Motion Visual Analogue Scale the Behavioral Inhibition/Behavioral Activation scale, the Affect Intensity Measure, the Emotional Intensity Scale, the Emotional Reactivity Scale, the Perth Emotional Reactivity Scale the Liebowitz Social Anxiety Scale, the Panic Disorder Severity Scale, the Penn State Worry Questionnaire, and the Systemizing Quotient. Each subject will also be asked to complete a Participant Satisfaction Survey. Subjects would complete this survey voluntarily and anonymously through a SurveyMonkey weblink.

**TES procedure:** In the course of our studies, we will use both sham controlled and open labeled study designs. Before each study, the subjects will be told in which study design they will be participating.

The stimulator for the study will be one of three devices, according to whether the stimulation will be done on-site or off-site. We also keep stimulators as back-up in case one device fails.

1. Transcranial Technologies (TCT): This device has programmable sham and real stimulation modes for transcranial direct current stimulation (tDCS). The appearance of the device is indistinguishable to the subject. In the sham stimulation mode, the device shows pseudorandom numbers on the screen that look like real stimulation is being delivered. The device gives a low level of stimulation at the very beginning and at the very end of the stimulation session in order to recreate the feeling of tingling that subjects perceive at the beginning and end of real stimulation. During real stimulation sessions, the device gives a real time measurement of resistance and change in resistance. These measurements are an indication of the adequacy of electrode contact with the scalp as well as dryness of the electrodes. If the resistance reaches a threshold set by the investigator, the device will automatically shut off. This will protect against skin burns. The current level used for the study will vary between 1-2mA. This low level of current can be powered by two 9V batteries.
2. The Pulvinar XCSITE 100: This device is able to provide both transcranial alternating current (tACS) and tDCS and is programmable for real and sham studies. It is designed and marketed for investigational research purposes by Pulvinar Neuro LLC. The device is not implanted has not been designed for or being used to support or sustain human life. This device does not have a potential for serious risk to the health, safety, or welfare of the participant. There has never been an instance of serious side-effect reported due to use of transcranial brain stimulation. This device may be used off-site as it is operated with a tablet with a user interface application (app). The device may be monitored via a WiFi connection in order to ensure that the participant has performed the stimulation appropriately. It is powered by a 9V battery. The device is equipped with 5 different stages of safety protection, all of which protect the stimulant from high currents. The stages are as follows:
3. Software parameter validation. All stimulation parameters are checked to be within the allowable ranges for those parameters. If values outside of this range are entered the app will not allow stimulation to start.
4. Automatic software current cutoff. The output of the current sensor described above is read by a microprocessor, which compares the reading to a value of +/- 2.3 mA peak. If the current exceeds these limits, stimulation is stopped, a relay in series with the electrode is opened, and the power supply used for stimulation is turned off. The user is then given the option to investigate the issue, and cancel or resume the test. Since high-side current sensing is used (described above), any short circuit of the electrode terminals to ground will be detected.
5. Automatic hardware current cutoff. The output of the current sensor is fed into a pair of comparators, which detect if the current exceeds +/- 4.5 mA. If so, the fault is latched such that the relay in series with the electrodes is opened. Additionally, the microprocessor is notified of this instance through an interrupt. Upon this interrupt, the microprocessor immediately stops stimulation and the power supply used for stimulation is turned off.
6. Permanent hardware current cutoff. A 5 mA fast-acting fuse is in series with the electrode connector. If the above over-current detection methods fail, the fuse will blow, and the participant will no longer be electrically connected to the current generator.
7. Power supply fuse. Finally, if for any other reason the entire device draws too much current, the main power supply fuse is blown. This fuse is rated for 400 mA, which is approximately 200% of nominal operating current.

3. NeuroConn DC-Stimulator: This device has programmable sham and real stimulation modes for double blind studies for tDCS and tACS. This device has been widely used in brain stimulation studies. As a safety feature, it provides a maximum current output of 2mA and has an internal battery that is charged through an outlet, but which cannot be run while being charged. If the resistance reaches a threshold set by the investigator, the device will automatically shut off. This will protect against skin burns. This is a non-portable device (insofar as it is too expensive to send home) that will be used on-site as a back-up stimulator.

The set-up for sham and real stimulation sessions are exactly the same. The investigator (or the subject after training) places an elastic band around their head. The electrodes are in sponges that are wet with saline (e.g.contact lens solution). Surface landmarks on the head or over the mastoid processes are used to place the electrodes, which are held in place by an elastic band. If the device is sent home, the setting cannot be changed except through password-protected access of the device. The stimulator ramps up the current over 30-60 seconds (time is set by the investigator). In the sham setting the current is ramped down again. In the real stimulation setting the current stays at the same level. The stimulation will proceed for 10-30 minutes at which point the current is slowly ramped down over 30-60 seconds. GVS sessions will be broken down into shorter sessions with no single session lasting more than 5-minutes, since it may cause some dizziness. The elastic band is removed and is placed in a plastic pouch along with the sponges. The device and accessories are returned to the case after each session.

For subjects who are treated on-site, we will also be performing EEG before and after the TES or GVS sessions.

**E. Statistical Methods**

Dependent measures will include questionnaire responses and clinical rating scales before and after TES sessions or overall treatment (which will be further factored into sham vs real TES if applicable). Dependent measures will be tabulated in spreadsheet form with distributions visualized and analyzed using STATA. Repeated measures ANOVA will be performed for difference scores between baseline and each subsequent time point for each set of questionnaires. Within-subject statistical models on each dependent measure with pre-TES and post-TES being the within-subject factor (and sham- vs real-TES as another within-subject factor if applicable) will be evaluated for the effect of TES on each outcome measure.

**F. Gender/Minority/Pediatric Inclusion for Research**

We will include participants age 18 and older. Women and minorities will be included in the study. Prior research indicates that MdDS predominantly affects women, without any predilection for any racial or ethnic groups.

**G. Human Participants**

We plan to enroll 300 subjects with MdDS and 50 healthy controls over a span of 10 years.

Inclusion criteria for MdDS subjects:

1. Age ≥18 years old
2. Willing and capable of interacting with the informed consent process
3. Primary disorder being a persistent perception of motion with no other cause determined after a careful interview.

4. Able to identity a study buddy and provide contact information

Inclusion criteria for healthy controls

1. Age ≥18 years old
2. Willing and capable of interacting with the informed consent process

Exclusion criteria for both:

1. Subjects who cannot comply with study conditions.

2. Active psychiatric condition such as mania or psychosis
3. Unstable medical condition
4. Implanted metal in the head or neck (metal or shrapnel, deep brain stimulators, aneurysm clips, cochlear stimulators, retinal implants, etc). Dental fillings are acceptable.

5. Any active skin disorder that affects skin integrity of the scalp.

6. History of any primary neurological disorder in the case of healthy controls

7. Pregnant or planning to become pregnant during the course of the study

Eligibility will be determined by the PI. Dr. Cha is a Board certified neurologist who has expertise in the evaluation of patients with motion perception disorders, auditory symptoms, movement disorders, pain, and stroke.

**H. Recruitment and Consent Procedure**

MdDS Subjects: Subjects will be recruited from advertisements: Flyers/Information Sheet/Internet Postings (www.mddsfoundation.org)

Screening will be conducted by study personnel in person, over the phone, or via a webcam portal such as Skype® or Facetime. A screening script will be used to ensure uniformity in the screening process. We will need to obtain their name and contact information should they pass the screening and still remain interested. If the subject is qualified by the initial phone screen, they will be invited to either an in-person or phone visit with the PI or study staff in order to go through the consent process. They will be emailed the consent form prior to the first visit in order for them to have adequate time to review and think of questions.

The informed consent process will include interactive assurances that participation in this study is voluntary, that there is no consequence to refusing to participate, that participating or not will not affect any ongoing or future care, and that the subject is free to withdraw at any time with no consequence to their ongoing or future care.

After the consent process, subjects will be invited to the research center for in-person training on the use of the TES devices. However, if they are unable to fly in for the procedure because of financial constraints, family obligations, etc. and have other compelling reasons not to be trained in person (eg. have a consistent history of having symptoms being worsened by travel), they will be given the option of receiving the training online through a webcam. If this option is chosen, the potential participant needs to be able to show that they have a reliable connection either through Skype or another live video streaming service, and that this connection will be available in a private setting. For example, the online training cannot be done with a webcam set-up in a common room with other people walking around. They will, however, be allowed to have a single study buddy who will help with the initial application of the device. Until the subject becomes accustomed to setting up the cap on him/herself, it can be helpful to have a second hand. The study buddy may also aid in communication between the PI and the participant, as well as to help send the stimulation device back to the PI. Information such as their name and contact information will be kept as a secondary contact for the participant. If the online training option is chosen, the device and accessories will be mailed to them ahead of the training session.

The subject will perform the very first treatment session with the PI observing, either in person or via webcam.

Before the actual treatment sessions are started, the subjects will complete the baseline questionnaires on SurveyMonkey, which will be repeated once a week for up to 24 weeks. They will keep a daily log of their sessions such as how many sessions they performed, for how long, and whether they had any side effects with each session. The side effects of the sessions are usually minor such as paresthesias, mild headache, and itching. These will be reported at the end of the week on their SurveyMonkey diary, which will be downloaded by the study staff each week and reviewed. However, they will have instructions that any side effects that are beyond these minor ones need to be reported to the PI that day and that the session should be terminated. At any point in the study, if the subjects feel that their symptoms are worsening or they do not want to continue for any reason (including no reason at all), they will stop their participation.

The SurveyMonkey weblink for our study will include a unique research portal URL. The data is transferred without SSL encryption. Since the subjects are identified through another means, there will never be any personally identifying information obtained through SurveyMonkey. They will only enter their study codes. The questionnaires will be sent via an email invitation that is scheduled according to the specific phase of the study. If the subject prefers to complete a paper diary, they will be given the option of doing so and will be given a booklet containing all the questionnaires that they will need for the study period.

Healthy Controls: These subjects will be recruited through an existing database of healthy control subjects at our institution who have expressed interest in participating in additional studies beyond the one through which they entered the database. They will undergo either a phone or in-person screen, depending on their preference. They will undergo the same questionnaires and screening interview as the MdDS participants.

**I. Subject Risks**

*Questionnaires:* *Risks and general protections against risk*

The main risk to the subjects is that questionnaires take time. The questionnaires being used in this study are the same as those used in our prior neuromodulation study, and the subjects report that they take no more than 30-minutes to complete. The priority of the data will be determined by the evolution of our study. Some people may find some of the questions to be too personal but the subjects always have the option of not answering any questions. The questionnaires will generally be accessed through a desktop computer but they can also be accessed through a smartphone or a tablet. If these portals cause symptom worsening, the subject will be given paper diaries.

*TES/GVS: Risks and general protections against risk*

Subjects will be fully informed about the foreseeable risks and discomforts associated with participation in this study. The consent forms describe these risks and discomforts clearly. Patients must know that they have the option to withdraw from TES/GVS studies at any time. Withdrawal from this study can be done without consequence. The investigators may also choose to terminate a participant from this study, if they suffer a severe adverse event, do not follow study requirements, or feel that continued participation would put the person at a greater risk than indicated.

*Specific protections against risk*
1. Headaches/itching/paresthesias. Headaches, itching, and paresthesias are generally very mild with TES therapy and are limited to the actual treatment duration. Since skin nerves habituate to the electrical stimulation rather quickly, most subjects are not aware of the stimulation after about the first 1-minute. This is what allows sham stimulation to be effectively masked. More persistent headache can be treated with acetaminophen or ibuprofen. The range of stimulation intensities for human studies of TES is usually 1-2mA. Our initial studies will begin with 1mA and will be gradually ramped up as tolerated by the subjects over subsequent studies.

2. Skin irritation. Skin redness is common with TES studies because the electrical stimulation increases local blood flow under the electrodes. This redness should dissipate within 30-minutes or less. The subjects will be instructed to observe the application area for redness before applying the electrodes each day. There should be no evidence of redness or skin breakdown before application. The subjects will also be asked to skip two days each week (ie only perform the stimulation for 5 of 7 days) in order to create a break in stimulation).

3. Dizziness. This can occur with the GVS stimulation at certain frequencies that have not been determined yet. The stimulation effects will be allowed to recover before starting a subsequent stimulation session. If the subject become dizzy, vertiginous, or develops a headache, the stimulation will be stopped.

**J. Benefits versus Risks**
In accordance to the principle of beneficence, research should maintain a favorable balance of benefit to risk. The proposed TES parameters in MdDS patients in this protocol fall within a Class II classification of benefit-risk ratio. That is, TES as used here is of potential, but unproven benefit. Given our experience, the FDA approval of a TES device that is actively marketed to the public, and experience of other TES laboratories, to date, the TES parameters used in this protocol are safe and constitute a ‘minimal risk’ application.

**K. Data and Safety Monitoring Plan**

The risk for adverse events is minimal. No medication, therapeutic decision or investigational device is made based on the results of these studies. Participants may experience some discomfort upon discussing their symptoms with staff and receiving unanticipated information about diagnosis. Participants may find it difficult to participate in detailed ratings. Any unanticipated adverse events will be reported immediately to the IRB of record and Laureate Institute for Brain Research Human Protection Administrator at (918) 502-5155 or via email at [hpa@laureateinstitute.org](mailto:hpa@laureateinstitute.org).

Each subject is given a unique identifier with a code. Information for each participant is entered into the Laureate Institute for Brain Research subject database and they are automatically given a LIBR ID e.g. AA001. The code key that links the unique identifier to the subjects’ names is kept in a separate file. All data analysis is performed on de-identified data.

Other than the principal investigator, there is no need for personally identifying information to be known to other investigators.

Data will be coded on two levels. Each subject will be given a unique code according to their primary assignment. When data is entered, it is entered in numerical form, eg (Yes=1, No=2). The data code is kept separate from the data, which would make the raw data un-interpretable otherwise.

Data that is entered through the online diaries through either SurveyMonkey or REDCap will be entered with the subject’s code, not their real name. No personally identifying information will be ascertained on the questionnaires. SurveyMonkey procedures allow for creation of a study specific research.net URL that only subjects within the study can ascertain. REDCap is an online electronic data capture program (project-redcap.org) used by over 600 institutions for anonymous data capture. As the study grows in scope, either one of these sites may be used. No personally identifying information is stored on either of these sites. The link to access the sites are, however, sent to the subject via their email address. If the subject does not consent to use of electronic data capture, they will always be given the option of entering all data on a paper document.

The link to the patient identifications as well as to the encoded data is stored on a password-protected computer on a separate spreadsheet in a locked room. Only Dr. Cha, the PI, will have access to the link. Anonymized data can be requested in writing to the PI. However, there would be no need for any personally identifying information to be revealed to other investigators.

If Dr. Cha leaves LIBR, and agreement will be made between LIBR and Dr. Cha’s new institution to transfer the data so that the study can continue. If another investigator at LIBR becomes a co-investigator of the study, their name will be added to the list of investigators and they will become responsible for maintaining data security at LIBR.

Study information will be made available to the subjects when there has been sufficient recruitment to make reasonable aggregate assessments. We can share general study characteristics (how many people were recruited, age, primary disorder etc.) early in the process but we do not share study results with subjects until the quality of the data is close to publication level.

**L. CONFIDENTIALITY**

Records of the participant’s participation in this study will be held confidential except as disclosure is required by law or as described in the informed consent document (under "Confidentiality"). The study doctor, the sponsor or persons working on behalf of the sponsor, and under certain circumstances, the United States Food and Drug Administration (FDA) and the Institutional Review Board (IRB) will be able to inspect and copy confidential study-related records which identify the subject by name. Therefore, absolute confidentiality cannot be guaranteed. If the results of this study are published or presented at meetings, the subject will not be identified.

Paper copies of consents, screening forms, the Research Privacy Form, and any other forms, testing results or papers containing Personally Identifiable Information (PII) will be stored in a secured medical records room with access granted only to authorized personnel.

**M: References**

**General MdDS**

1. Cha YH. Mal de Debarquement. Semin Neurol. 2009;29 (5):520-7

2. Cha YH, Brodsky J, Ishiyama G, Sabatti C, Baloh RW. Clinical features and associated syndromes of mal de debarquement. J Neurol 2008;255:1038-1044.

3. Hain TC, Hanna PA, Rheinberger MA. Mal de Debarquement. Arch Otolaryngol Head Neck Surg 1999;125:615-620.

4. Gordon CR, Spitzer O, Doweck I, Melamed Y, Shupak A. Clinical features of Mal de Debarquement: adaptation and habituation to sea conditions. J Vest Res 1995;5:363-369.

5. Brown JJ, Baloh RW. Persistent mal de debarquement: a motion induced subjective disorder of balance. Am J Otolaryngol 1987;8:219-222.

6. Cha YH, Chakrapani S, Craig A, and Baloh RW. Metabolic and functional connectivity changes in mal de debarquement syndrome. PLoS One. 2012;7(11):e49560.

7. Cha YH, Cui Y, and Baloh RW. Repetitive transcranial magnetic stimulation for mal de debarquement syndrome. Otol Neurotol. 2013, Jan;34(1):175-9.

**General TES/GVS Safety**

1. Brunoni AR, Amadera J, Berbel B, Volz MS, Rizzerio BG, and Fregni F. A systematic review on reporting and assessment of adverse effects associated with transcranial direct current stimulation. Int J Neuropsychopharmacol. 2011, Sep;14(8):1133-45.
2. Brunoni AR, Nitsche MA, Bolognini N, Bikson M, Wagner T, Merabet L, et al. Clinical research with transcranial direct current stimulation (tDCS): challenges and future directions. Brain Stimul. 2012, Jul;5(3):175-95.
3. Poreisz C, Boros K, Antal A, and Paulus W. Safety aspects of transcranial direct current stimulation concerning healthy subjects and patients. Brain Res Bull. 2007, May 30;72(4-6):208-14.
4. Loo CK, Martin DM, Alonzo A, Gandevia S, Mitchell PB, and Sachdev P. Avoiding skin burns with transcranial direct current stimulation: preliminary considerations. The International Journal of Neuropsychopharmacology. 2011;14(03):425-426.
5. Frank E, Wilfurth S, Landgrebe M, Eichhammer P, Hajak G, and Langguth B. Anodal skin lesions after treatment with transcranial direct current stimulation. BRAIN STIMULATION: Basic, Translational, and Clinical Research in Neuromodulation. 2010;3(1):58-59.
6. Utz K, Dimova V, Oppenlander K, Kerkhoff G. Electrified minds: Transcranial direct current stimulation (tDCS) and Galvanic Vestibular Stimulation (GVS) as methods of non-invasive brain stimulation in neuropsychology-A review of current data and future implications. Neuropsychologia, 2010; 48(10): 2789-2810.
7. Cha YH, Urbano D, Pariseau N. Randomized single blind sham controlled trial of adjunctive home-based tDCS after rTMS for Mal de Debarquement Syndrome: safety, efficacy, and participant satisfaction assessment. Brain Stimulation 2016; 9(4): 537-544.

**General TES Therapy**

1. George MS, Padberg F, Schlaepfer TE, O'Reardon JP, Fitzgerald PB, Nahas ZH, and Marcolin MA. Controversy: Repetitive transcranial magnetic stimulation or transcranial direct current stimulation shows efficacy in treating psychiatric diseases (depression, mania, schizophrenia, obsessive-complusive disorder, panic, posttraumatic stress disorder). Brain Stimul. 2009, Jan;2(1):14-21.
2. Berlim MT, Van den Eynde F, and Daskalakis ZJ. Clinical utility of transcranial direct current stimulation (tDCS) for treating major depression: a systematic review and meta-analysis of randomized, double-blind and sham-controlled trials. J Psychiatr Res. 2013, Jan;47(1):1-7.
3. Song JJ, Vanneste S, Van de Heyning P, and De Ridder D. Transcranial direct current stimulation in tinnitus patients: a systemic review and meta-analysis. ScientificWorldJournal. 2012;2012427941.
4. Fregni F, Freedman S, and Pascual-Leone A. Recent advances in the treatment of chronic pain with non-invasive brain stimulation techniques. Lancet Neurol. 2007, Feb;6(2):188-91.
5. Schlaug G, and Renga V. Transcranial direct current stimulation: a noninvasive tool to facilitate stroke recovery. Expert Rev Med Devices. 2008, Nov;5(6):759-68.
6. McCreery DB, Agnew WF, Yuen TG, and Bullara L. Charge density and charge per phase as cofactors in neural injury induced by electrical stimulation. Biomedical Engineering, IEEE Transactions on. 1990;37(10):996-1001.
7. Benninger DH, Lomarev M, Lopez G, Wassermann EM, Li X, Considine E, and Hallett M. Transcranial direct current stimulation for the treatment of Parkinson's disease. Journal of Neurology, Neurosurgery & Psychiatry. 2010;81(10):1105-1111.
8. Ayache SS, Farhat WH, Zouari HG, Hosseini H, Mylius V, and Lefaucheur JP. Stroke rehabilitation using noninvasive cortical stimulation: motor deficit. Expert Rev Neurother. 2012, Aug;12(8):949-72.
