# Supplementary material for: Correction: Double-blind randomized N-of-1 trial of transcranial alternating current stimulation for mal de débarquement syndrome
Source: PLoS One. 2024 Dec 10;19(12):e0315783. doi: 10.1371/journal.pone.0315783 (PMC11630578; doi:10.1371/journal.pone.0315783)
Supplement: S2 File — (PDF) [file pone.0315783.s002.pdf]

PLOS ONE

RESEARCH ARTICLE

Double-blind randomized N-of-1 trial of transcranial alternating current stimulation for mal de débarquement syndrome

Yoon-Hee Cha[1,2]*, Diamond Gleghorn[3], Benjamin Chipper Doudican[1]

1 University of Minnesota, Minneapolis, MN, United States of America, **2** Laureate Institute for Brain Research, Tulsa, OK, United States of America, **3** Missouri State University, Springfield, MO, United States of America

* ycha@umn.edu

Check for updates

OPEN ACCESS

Citation: Cha Y-H, Gleghorn D, Doudican BC (2022) Double-blind randomized N-of-1 trial of transcranial alternating current stimulation for mal de débarquement syndrome. PLoS ONE 17(2): e0263558. https://doi.org/10.1371/journal.pone.0263558

Editor: Sandra Carvalho, University of Aveiro Department of Education and Psychology: Universidade de Aveiro Departamento de Educacao e Psicologia, PORTUGAL

Received: September 4, 2020

Accepted: January 13, 2022

Published: February 4, 2022

Copyright: © 2022 Cha et al. This is an open access article distributed under the terms of the Creative Commons Attribution License, which permits unrestricted use, distribution, and reproduction in any medium, provided the original author and source are credited.

Data Availability Statement: All data files are available from the Harvard Dataverse database: https://doi.org/10.7910/DVN/FITEYY.

Funding: This work was supported by the Laureate Institute for Brain Research, the William K. Warren Foundation, the MdDS Balance Disorders Foundation, the Springbank Foundation (YHC), NSF EPSCoR RII Track-2 #1539068 (YHC, and NIH/NIGMS grant P20 GM121312 (YHC). The

Abstract

Background

Mal de Débarquement Syndrome (MdDS) is a medically refractory neurotological disorder characterized by persistent oscillating vertigo that follows a period of entrainment to oscillating motion such as experienced during sea or air travel. Fronto-occipital hypersynchrony may correlate with MdDS symptom severity.

Materials and methods

Individuals with treatment refractory MdDS lasting at least 6 months received single administrations of three fronto-occipital transcranial alternating current stimulation (tACS) protocols in an "n-of-1" double-blind randomized design: alpha frequency anti-phase, alpha-frequency in-phase, and gamma frequency control. Baseline assessments were made on Day 1. The treatment protocol that led to the most acute reduction in symptoms during a test session on Day 2 was administered for 10–12 stacked sessions given on Days 3 through 5 (20-minutes at 2-4mA). Pre to post symptom changes were assessed on Day 1 and Day 5. Participants who could clearly choose a preferred protocol on Day 2 did better on Day 5 than those who could not make a short-term determination on Day 2 and either chose a protocol based on minimized side effects or were randomized to one of the three protocols. In addition, weekly symptom assessments were made for four baseline and seven post stimulation points for the Dizziness Handicap Inventory (DHI), MdDS Balance Rating Scale (MBRS), and Hospital Anxiety and Depression Scale (HADS).

Results

Of 24 participants, 13 chose anti-phase, 7 chose in-phase, and 4 chose control stimulation. Compared to baseline, 10/24 completers noted $\geq$ 25% reduction, 5/24 $\geq$50% reduction, and 2/24 $\geq$75% reduction in oscillating vertigo intensity from Day 1 to Day 5. Stimulating at a frequency slightly higher than the individual alpha frequency (IAF) was better than stimulating at exactly the IAF, and slightly better than stimulating with a strategy of standardized

funders had no role in study design, data collection and analysis, decision to publish, or preparation of the manuscript.

Competing interests: The authors have declared that no competing interests exist.

stimulation at 10Hz. A one-way repeated measures ANOVA of weekly DHI, MBRS, and HADS measurements showed significant reductions immediately after treatment with improvement increasing through post-treatment week 6.

Conclusion

Fronto-occipital tACS may be effective in reducing the oscillating vertigo of MdDS and serve as a portable neuromodulation alternative for longer-term treatment. Stimulation frequency relative to the IAF may be important in determining the optimum treatment protocol [ClinicalTrials.gov study NCT02540616. https://clinicaltrials.gov/ct2/show/NCT02540616].

Introduction

Mal de débarquement Syndrome (MdDS) is a neurotological disorder resulting from entrainment to oscillating motion, such as occurs during sea or air travel [1,2]. MdDS symptoms include persistent oscillating vertigo, fatigue, cognitive slowing, visual motion intolerance, and headaches [3–5]. The vertigo of MdDS is often described as a rocking, bobbing, or swaying feeling that only nulls with re-exposure to passive motion [6]. Unlike landsickness, which is a brief period of motion-induced oscillating vertigo that lasts for less than 48-hours and is common among healthy individuals, MdDS, can persist for months or years; this can lead to significant morbidity [6–8]. Medications remain palliative and treatment options for MdDS remain limited, particularly ones that do not require travel or high out-of-pocket costs.

Biological markers for the MdDS brain-state include differences in both long-range cortico-cortical connectivity and neocortical-limbic connectivity [9–11]. In particular, fronto-occipital connectivity in the alpha frequency correlates with symptom modulation after non-invasive brain stimulation with repetitive transcranial magnetic stimulation (rTMS) [9,10,12]. Decreasing posterior default mode connectivity with the entorhinal cortex correlates with reduction in vertigo intensity [13].

In prior studies, reduction in oscillating vertigo after rTMS over the dorsolateral prefrontal cortex (DLPFC) correlated with increased functional connectivity in the low alpha (8-10Hz) band but decreased connectivity in the high alpha (10-13Hz), beta (14-30Hz), and gamma bands (>30Hz) [9,12]. The inflection point at which increasing versus decreasing connectivity in the alpha band is beneficial was noted on group level analyses; it is not known what that inflection point is for an individual. It remains to be determined, therefore, whether individual factors, such as the individual alpha frequency (IAF) should be considered in treatment designs for MdDS or whether a standard treatment frequency is sufficient. The IAF is the frequency within the alpha band (8-13Hz) that has the strongest power. It varies between individuals but is heritable and stable within an individual [14,15]. Individual alpha frequency correlates with aging and cognitive demands and may thus be relevant for symptoms that are affected by these factors [16,17].

We explored whether the connectivity modulations induced indirectly through rTMS might be directly achievable by entraining fronto-occipital networks with transcranial alternating current stimulation (tACS) and lead to persistent treatment response after a period of stimulation. tACS is a form of non-invasive brain stimulation in which low levels of current are applied to the scalp at a frequency that is tuned to entrain underlying latent rhythms [18–20]. This method may be used to increase or decrease functional connectivity or change the power of specific frequency bands. Individuals with MdDS have hypersynchrony revealed through

greater 40-Hz power of the auditory steady-state response (ASSR), which decreases as a function of improved symptoms after fronto-occipital tACS, at least in the short-term [21]. If improvement in symptoms can last beyond induction treatment, tACS may become a less expensive and portable alternative to other forms of neuromodulation that require travel to obtain.

The present study addressed the immediate and six-week clinical outcome of treatment of MdDS with tACS. The study used an "n-of-1," design to determine which pattern and frequency of stimulation relative to the participants' IAF was associated with the greatest reduction in intensity of oscillating vertigo. By administering two different tACS paradigms that induced opposite connectivity effects (synchronizing vs desynchronizing) with the addition of a control condition, we determined whether there was an association between the participant's IAF and the most effective treatment paradigm. The design is similar to a prior study using continuous theta burst stimulation (cTBS) that had employed two real and one control condition in a similar "n-of-1," design [22]. In these designs, the participants are given three different treatment protocols in a double-blind randomized order and are ultimately treated with the individually most effective protocol based on short-term responses after each protocol. Each treatment protocol was labeled as "1", "2", and "3" with the assignments only known to the research staff administering the sessions but not to the principal investigator or anyone performing the data analysis. The identity of the individually chosen protocols were unblinded at the conclusion of the study. Ethically, the study design could not include a true randomization to a control arm since MdDS is a travel-induced disorder and all participants travel from long distances to participate in these studies. It was previously shown that administering true control (sham) stimulation to individuals with MdDS who travel to participate in clinical trials was associated with worsened symptoms [23]. This design, in which the participants themselves could choose the control condition if it was perceived to be the most effective, avoided this ethical issue.

Materials and methods

Informed consent

Study procedures were completed according to Declaration of Helsinki guidelines and approved by Western IRB (www.wirb.com). Participants provided written informed consent and were recruited under ClinicalTrials.gov study NCT02540616, which recruits for several different transcranial electrical current studies. This study used tACS in an off-label manner and was completed between July 2017 and June 2019 at the Laureate Institute for Brain Research in Tulsa, OK. in a laboratory setting.

Inclusion and exclusion criteria

Inclusion criteria included: 1. Persistent oscillating vertigo that started within 48-hours after disembarking from sea, air, or land-based travel, 2. Symptom duration of at least six months, 3. No other cause for symptoms after evaluation by a neurologist or otolaryngologist with appropriate testing for peripheral inner ear or other central nervous system cause for symptoms. Exclusion criteria included: 1. An unstable medical or psychiatric condition, including bipolar disorder or any cause for psychosis, 2. Pregnant or planning to become pregnant during the study, 3. Contraindications to undergoing tACS, EEG, fMRI, including skin disorders (note, this study included EEG and fMRI but those data are included in separate reports), 4. An unclear history of the onset of symptoms, 5. An inability to complete all study related testing. Exclusion criteria were determined for participant safety and to minimize fluctuations in symptoms that were not related to treatment effects. The study recruited a medically refractory

population that had tried and failed at least one benzodiazepine, a selective serotonin reuptake inhibitor (SSRI) or a SSRI/selective norepinephrine reuptake inhibitor (SNRI), and physical therapy. The CONSORT diagram of the recruitment pathway for this study is shown in Fig 1.

Trial design

This was a double-blind "n-of-1," study. Both the principal investigator and the participants were blinded to the order of each parameter administered until the conclusion of the study. The participants underwent consenting procedures, interviews, and baseline EEG and fMRI studies on Day 1 (Fig 2). On Day 2, the participants received one session each of three stimulation protocols given in randomized order between participants: 1] Anti-phase (desynchronizing) alpha frequency stimulation, 2] In-phase (synchronizing) alpha frequency stimulation, 3] Anti-phase gamma frequency (40Hz) control stimulation. The order of stimulation was determined at the beginning of the study with each stimulation order given sequentially according to the order of participant entry. One research assistant created the order of stimulation and

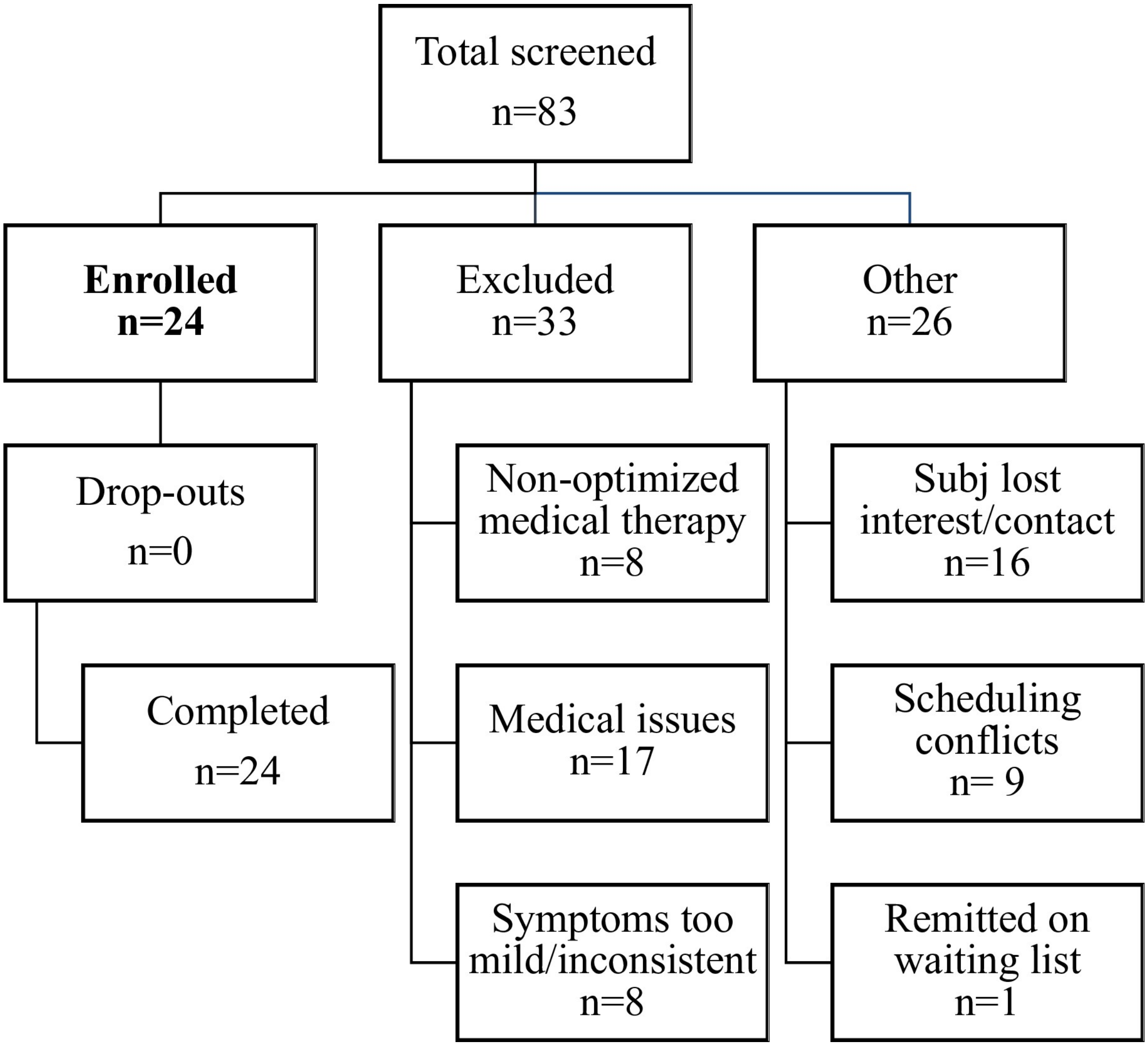

Fig 1. CONSORT diagram for recruitment.

https://doi.org/10.1371/journal.pone.0263558.g001

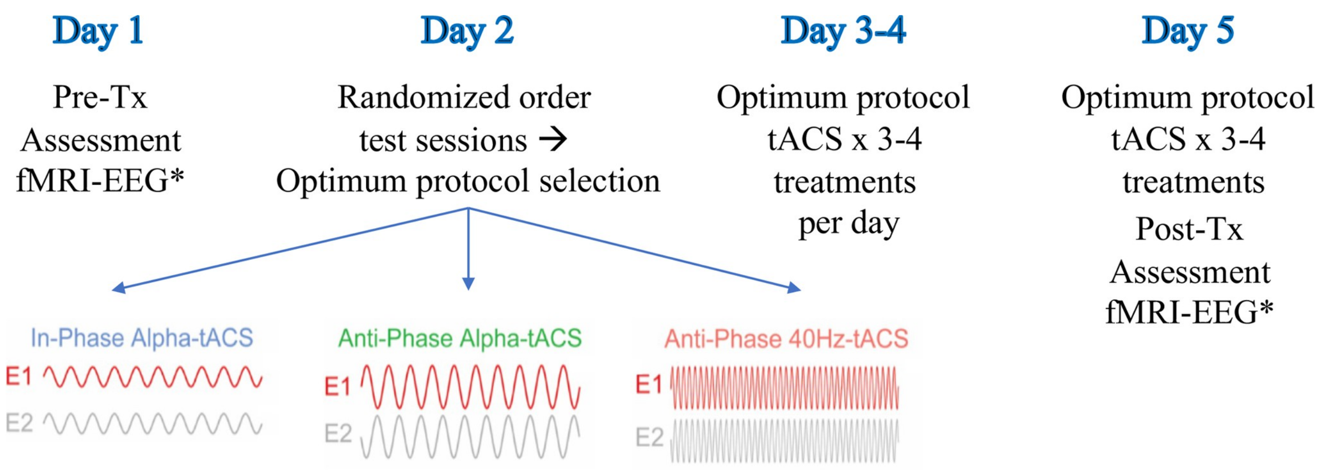

Fig 2. Study procedures Day 1 through Day 5. Baseline clinical and symptom assessments were performed in the morning with fMRI and EEG obtained in the afternoon of Day 1. Day 2 entailed test sessions of each of three protocols (anti-phase, in-phase, control) given in an unlabeled and randomized order. Participants elected their individually most effective protocol. Individually chosen optimal treatments were given on the mornings of Days 3 and 4. Final treatments were given on the morning of Day 5 followed by fMRI and EEG in the afternoon.

https://doi.org/10.1371/journal.pone.0263558.g002

another administered the sessions. Each order of stimulation (six possible orders given three protocols) was given to four individuals, which resulted in a cohort of 24 participants. Each stimulation was referred to as "1," "2," or "3," during the trial. Since tACS has never been performed in MdDS previously, a pre-defined sample size to detect effect size could not be determined. This was a pilot study to assess initial treatment effects and was not powered to detect differences between treatment protocols.

tACS procedures

tACS was performed with the Pulvinar XCSITE 100 stimulator (https://www.pulvinarneuro.com) at 2mA for anti-phase alpha and control stimulation and 4mA for in-phase stimulation. Stimulation was through two 10cmx10cm sponge electrodes with one placed on the forehead above the eyebrow line and the other placed over the occipital region above the inion (**Fig 3**). The sponges housed carbonized rubber electrodes, were wet with commercial normal saline, and were snapped into custom-fit neoprene headbands so that they were placed over the same location in every stimulation session. The electrodes were held in place with additional pressure applied through elastic bands. Care was taken to avoid causing wetness of the headbands.

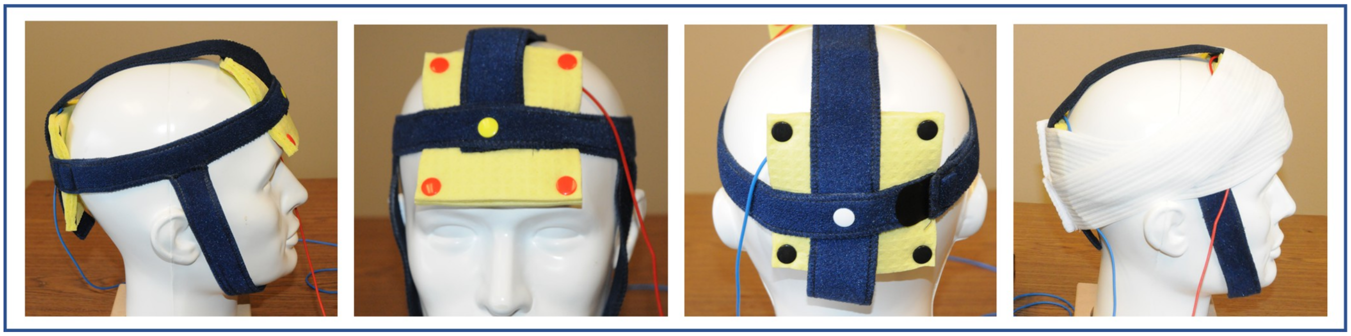

Fig 3. Model of tACS montage used in study. 10x10cm sponges housing carbonized rubber electrodes were placed over the frontal pole above the eyebrows and above the inion and held down with neoprene straps and headbands. Care was taken to avoid causing wetness of the headbands.

https://doi.org/10.1371/journal.pone.0263558.g003

The anti-phase alpha and control conditions used two cranial electrodes. For the in-phase condition, the current to the two cranial electrodes was split with a cable splitter. The return electrode was placed on the left upper arm. Each stimulation session lasted 20-minutes.

During the tACS administration, participants were seated in a recliner facing a window in order to mask out the phosphenes that were generated with the stimulation. Stimulation sessions were performed with eyes closed. After each stimulation session was completed, the participants reported the change in the intensity of their oscillating vertigo intensity for 60-minutes on a scale of 0–100 in which 0 represented no vertigo and 100 represented vertigo so severe that standing was not possible. The stimulation protocol that most optimally lowered the participant's symptoms on the VAS and which improved balance was chosen for repetitive treatment sessions administered on Days 3 to 5 (**Fig 2**). Balance was assessed with the modified Balance Error Scoring System (mBESS) using the SWAY Balance® mobile app on an iPod Touch [22,24]. If there was no clearly beneficial protocol, the participant chose the paradigm that was best tolerated. No participant had to stop any sessions. However, if they were unsure of which protocol was best, then two protocols could be repeated on Day 3 in the reverse order in which they were initially given on Day 2. The participant then selected the best protocol. Therefore, some participants received 10 back-to-back treatments with the same protocol while others received 12 back-to-back treatments.

For the initial 12 participants who were enrolled in the study, the alpha-frequency stimulation was given at 10Hz. Stimulation device capabilities of the Pulvinar device increased during the course of the study, which allowed progressively more precise stimulation frequencies within 0.1Hz increments. This allowed us to study the difference in outcomes of stimulating at the IAF (n = 6) and slightly higher than the IAF (n = 6). Since the IAF can be different at different electrode positions, the IAF at EEG electrode position Oz was used.

All stimulation sessions were started in the morning between 8am-9am and in general completed by 1pm. As time allowed, the participants were treated with three to four sessions a day for three days, culminating in 10–12 sessions per participant. Each 20-minute stacked treatment session was separated by at least a 30-minute break in between sessions. The participants traveled home the day after the last treatment on Day 5 and were advised of travel precautions. They were required to avoid any medication changes or travel lasting more than two-hours for the duration of the reporting period of the study.

Reporting

Symptom reporting for this study was exactly the same as for prior neuromodulation studies on MdDS [22,25]. Participants completed weekly questionnaires with individualized participant codes on an encrypted SurveyMonkey® weblink. Reports started three weeks before and commenced six weeks after their on-site visit, which created four sets of pre-stimulation questionnaires, one immediate treatment questionnaire, and six sets of post stimulation questionnaires (total 11 sets of data). The questionnaires included the Dizziness Handicap Inventory (DHI) [26] scored from 0–100; the MdDS Balance Rating Scale (MBRS) [22,25] scored from 1–10; and the Hospital Anxiety and Depression Scale (HADS) [27] with each component scored from 0–21. Higher scores represent worse symptoms on all questionnaires. Participants were compensated at our standard institutional rates for each study component, which included interviews, tACS sessions, fMRI and EEG as well as for the online diaries. They were not reimbursed for travel expenses.

Statistical analysis

Data were analyzed with STATA IC version 14.2 (www.stata.com) using VAS scores measured between Day 1 and Day 5 and percent change in vertigo intensity measured with the Day 1

score as the baseline. The median of the four pre-treatment weekly scores for the DHI, MBRS, and HADS were used as the baseline with the change from this baseline calculated for the seven post-treatment scores. These difference scores were entered into a one-way repeated-measures ANOVA analysis. Greenhouse-Geisser correction was made to account for the non-independence of within subject data. Linear prediction models with 95% confidence intervals were calculated for the DHI, MBRS, HADS Anxiety, and HADS Depression scores.

Results

Participant characteristics

Twenty-two right-handed and two left-handed tACS naïve individuals (23 women, one man) with MdDS were recruited. Mean age at the time of the study was 53.0± 11.8 years (range: 22–66 years, median: 57.0 years) and mean duration of illness = 38.6±53.4 months (range: 6–240 months, median: 18.0 months). Triggers included 15 water travel, nine air travel, and one land travel (Table 1). There was one participant who could not distinguish between water and air as her trigger so was counted in both groups.

All twenty-four participants completed 10–12 sessions of tACS given over three days. Upon unblinding the study, it was determined that eight participants had chosen anti-phase alpha tACS, six had chosen in-phase alpha tACS, and two had chosen the anti-phase gamma control

Table 1. Demographic data of study participants.

Participant	Age at Study	Handedness	Duration of illness in months	Trigger	Benzodiazepine	SSRI	Distance (mi)
1	57	R	13	Plane	No	No	1072
2	65	R	120	Cruise	Yes	No	220
3	36	R	22	Plane	Yes	Yes	463
4	60	R	21	Plane	Yes	No	722
5	60	R	14	Boat	No	No	691
6	64	R	51	Boat	Yes	No	1605
7	53	R	8	Boat	No	Yes	650
8	57	R	55	Flight+Roller Coaster	No	Yes	1282
9	39	L	41	Cruise	No	No	1166
10	57	R	240	Boat	No	Yes	14
11	57	L	11	Cruise	No	No	1595
12	63	R	8	Cruise	No	No	106
13	61	R	38	Swaying tower	No	No	2020
14	66	R	55	Cruise	No	No	1667
15	29	R	22	Plane	No	Yes	118
16	53	R	14	Lake swimming	No	No	1439
17	57	R	16	Plane	No	No	1216
18	57	R	120	Plane, boating	Yes	No	746
19	66	R	20	Cruise	Yes	Yes	1728
20	47	R	9	Cruise	No	Yes	452
21	57	R	6	Boat	No	No	640
22	49	R	6	Cruise	No	No	756
23	41	R	8	Plane+Amusement Park	No	No	531
24	22	R	8	Plane	Yes	No	658

Under the SSRI column, participants who were using a mixed SSRI/SNRI are indicated with an *. The distance column indicates the number of miles between the participant's place of origin and the study site.

https://doi.org/10.1371/journal.pone.0263558.t001

as the clearly the best protocol. There were an additional eight participants who had indicated that there was no difference between the three protocols in terms of vertigo reduction. These eight were treated with the protocol that was the best tolerated, i.e. that the participant found least unpleasant. In general, the participants found the in-phase protocol to be the most unpleasant because of paresthesias induced under the electrode on the arm used in this montage. Therefore, they chose one of the two anti-phase protocols. This culminated in 13 total participants being treated with anti-phase alpha, seven with in-phase alpha, and four with anti-phase gamma control (S1 Fig). The first 12 participants were treated at 10Hz alpha frequency. The next 12 participants were randomized to be treated at either a frequency 0.1–0.4Hz above their IAF or exactly at their IAF.

Short-term response

Thirteen of 24 (54%) participants reported ≥10-points, 9/24 (36%) ≥20-points, and 3/24 (13%) ≥30-point reduction in symptom scores on the 0–100 VAS. This corresponded to 10/24 (42%) reporting ≥25% reduction, 5/24 (21%) reporting ≥50% reduction and 2/24 (8%) reporting ≥75% reduction in vertigo intensity from Day 1 to Day 5 (Fig 4). A small number of participants worsened during the week but no participant worsened by more than 10-points. The one individual who worsened by 10-points had chosen the gamma frequency control condition (the other three who had chosen the control condition experienced 0, -5, and -10-points of symptom change). There was a suggestion that stimulation at exactly IAF was not as effective as stimulation above the IAF or at 10Hz (Fig 5). Stimulation sessions were well-tolerated with the main complaints being of paresthesias on the head and arm under the electrodes, the generation of phosphenes, and fatigue. These effects were expected; no stimulation session had to be stopped because of discomfort. Individual responses by protocol and strategy are listed in Table 2. Participants who were able to clearly select a preferred treatment protocol on Day

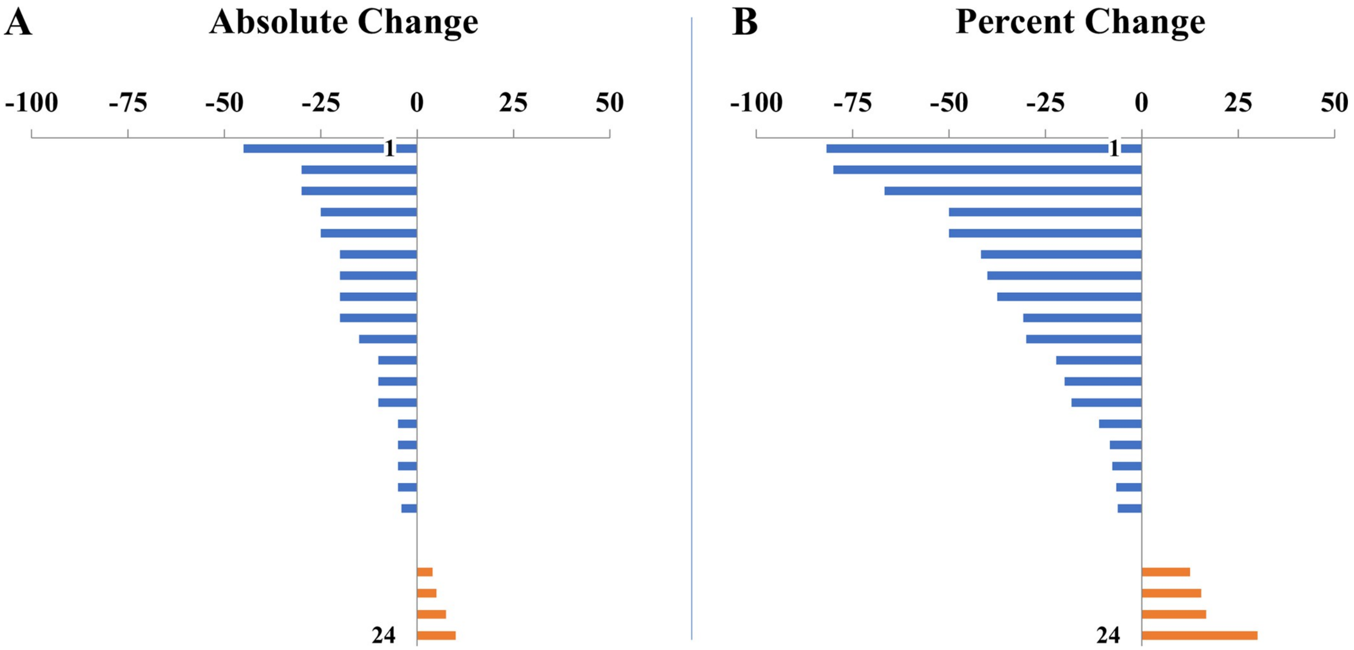

Fig 4. Total treatment response to tACS. (A) Absolute change and (B) Percent change in symptoms after 10–12 sessions of the tACS paradigm of the participant's choice. Scores were calculated based on a 0–100 Visual Analogue Scale in which lower values represent lower symptom severity.

https://doi.org/10.1371/journal.pone.0263558.g004

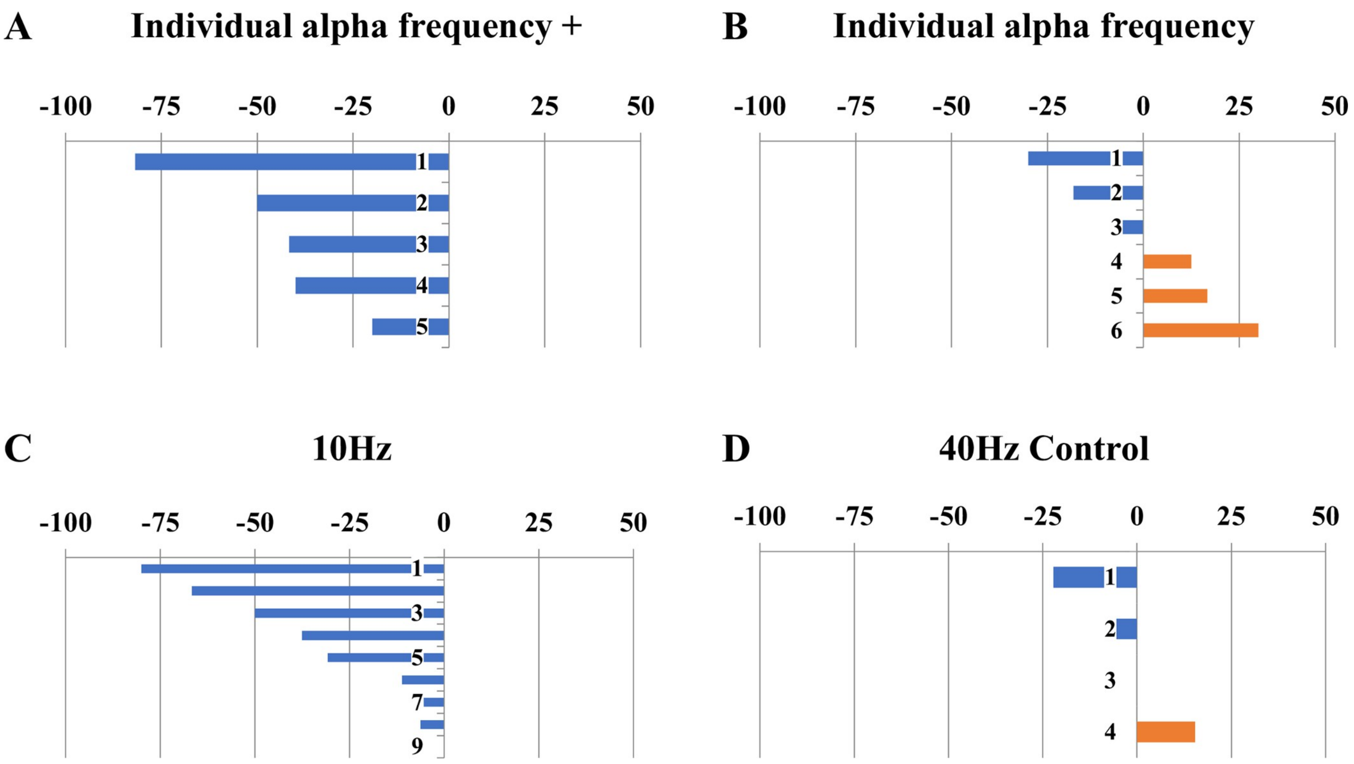

Fig 5. Percentage treatment response by stimulation type. Percentage change in symptoms after 10–12 sessions of alpha tACS according to type of stimulation used: (A) higher than IAF, (B) IAF, (C) standard 10Hz, (D) standard 40Hz. These figures combine both anti-phase and in-phase stimulation.

https://doi.org/10.1371/journal.pone.0263558.g005

2 had higher treatment responses by Day 5 compared to those who could not determine an optimal protocol (Mean change -32.8+/-30.4 vs -1.3+/-13.2, two-tailed $p<0.005$).

Post-stimulation response

Repeated measures ANOVA using the four-week pre-treatment median scores for DHI, MBRS, and HADS as the baseline showed significant decreases in DHI [F(10,23) = 6.24, *p*<0.0.001], MBRS [F(10,23) = 3.23, *p*<0.01], the HADS Anxiety sub-score [F(10,23) = 11.40, *p*<0.001], and HADS Depression sub-scores [F(10,23) = 4.04, *p*<0.005] during the course of the study. Linear prediction models with 95% confidence intervals showed score changes decreasing significantly ($p<0.05$) for six weeks post tACS for all measures (Fig 6).

Discussion

We report results of an "n-of-1" design trial of fronto-occipital tACS for medically refractory MdDS. Choice of in-phase (synchronizing) alpha and anti-phase (desynchronizing) alpha stimulation were comparable but anti-phase stimulation was preferred when considering side effects and comfort. There was a suggestion that stimulation at slightly above the IAF was better than stimulation at the IAF. Though the number of participants in each group was small, these findings indicate that knowledge of the IAF could be helpful in optimizing tACS treatment paradigms for MdDS. Participants who were able to clearly choose a preferred treatment protocol on the Day 2 testing day had significantly better reduction in symptoms after multiple sessions than those who could not choose a preferred protocol, suggesting that individual tailoring of treatment protocols through an n-of-1 design could have some predictive value of longer-term treatment response.

Table 2. Individual treatment response by protocol, frequency relative to IAF, and initial participant choice.

Participant	Protocol used	Strategy	IAF at Oz (Hz)	Stim frequency (Hz)	Stim frequency relative to IAF	Day 1 VAS	Day 5 VAS	Day 5-Day 1 VAS change	Percent VAS change	Participant choice on Day 2
1	Anti-phase	10Hz	9.6	10	0.4	25	5	-20	-80	2
2	Anti-phase	10Hz	10.4	10	-0.4	30	10	-20	-67	2
3	Anti-phase	IAF+	8.6	9	0.4	50	25	-25	-50	2
4	Anti-phase	10Hz	8.7	10	1.3	60	30	-30	-50	2
5	Anti-phase	IAF+	10.4	10.5	0.1	60	35	-25	-42	2
6	Anti-phase	IAF	10.1	10.1	0	50	35	-15	-30	2
7	Anti-phase	IAF+	9.6	10	0.4	50	40	-10	-20	2
8	Anti-phase	IAF	11.2	11.2	0	60	55	-5	-8	None
9	Anti-phase	10Hz	8.7	10	1.3	75	70	-5	-7	None
10	Anti-phase	10Hz	9.3	10	0.7	64	60	-4	-6	None
11	Anti-phase	10Hz	8.8	10	1.2	80	80	0	0	None
12	Anti-phase	IAF	8.6	8.6	0	32	36	4	13	2
13	Anti-phase	IAF	9	9	0	25	32.5	7.5	30	None
14	In-phase	IAF+	8.3	8.5	0.2	55	10	-45	-82	1
15	In-phase	IAF+	7.8	8	0.2	50	30	-20	-40	1
16	In-phase	10Hz	8.8	10	1.2	80	50	-30	-38	1
17	In-phase	10Hz	10.4	10	-0.4	65	45	-20	-31	1
18	In-phase	IAF	11.4	11.4	0	55	45	-10	-18	1
19	In-phase	10Hz	7.9	10	2.1	45	40	-5	-11	None
20	In-phase	IAF	11.4	11.4	0	30	35	5	17	1
21	Control	10Hz	9	40	>30	45	35	-10	-22	3
22	Control	10Hz	8.1	40	>30	65	60	-5	-8	None
23	Control	10Hz	9.7	40	>30	70	70	0	0	None
24	Control	IAF+	8.9	40	>30	65	75	10	15	3

Note: Participant number on Table 2 does not correlate with the number on Table 1. Data in Table 2 are organized according to treatment protocol and then by magnitude of treatment response. Choice 1 = in-phase, 2 = anti-phase, 3 = control.

https://doi.org/10.1371/journal.pone.0263558.t002

We used fronto-occipital stimulation in this study because EEG based functional connectivity measurements have shown that long-range fronto-occipital connectivity changes as a function of symptom modulation of the oscillating vertigo experienced in MdDS [9,12,13]. In our prior work, increase in functional connectivity in the low alpha band (8-10Hz) correlated with symptom improvement after rTMS over prefrontal cortex [9,12]. This would predict that in-phase stimulation, which increases functional connectivity, should be more effective at lower stimulation frequencies. However, decreasing functional connectivity at higher frequencies (high alpha band and higher), was also associated with symptom improvement. Where the transition point is in each individual was not clearly related to the choice of in-phase versus anti-phase stimulation in this study though stimulation at a frequency higher than the IAF seemed to be more beneficial. Therefore, in any individual, therapies may need to be tailored to individual responses with the IAF as a marker.

In comparison to the response to cTBS over occipital cortex and cerebellum, the acute improvements to tACS were not as dramatic [22] (**S2 Fig**). However, the long-term effects were comparable to cTBS and somewhat better than prefrontal rTMS [25] (**S3 Fig**). Though the effect on any individual was not predictable, the group-level rebound worsening of symptoms after travel home that was observed after both the cTBS and prefrontal rTMS paradigms was not observed in this study [22,25].

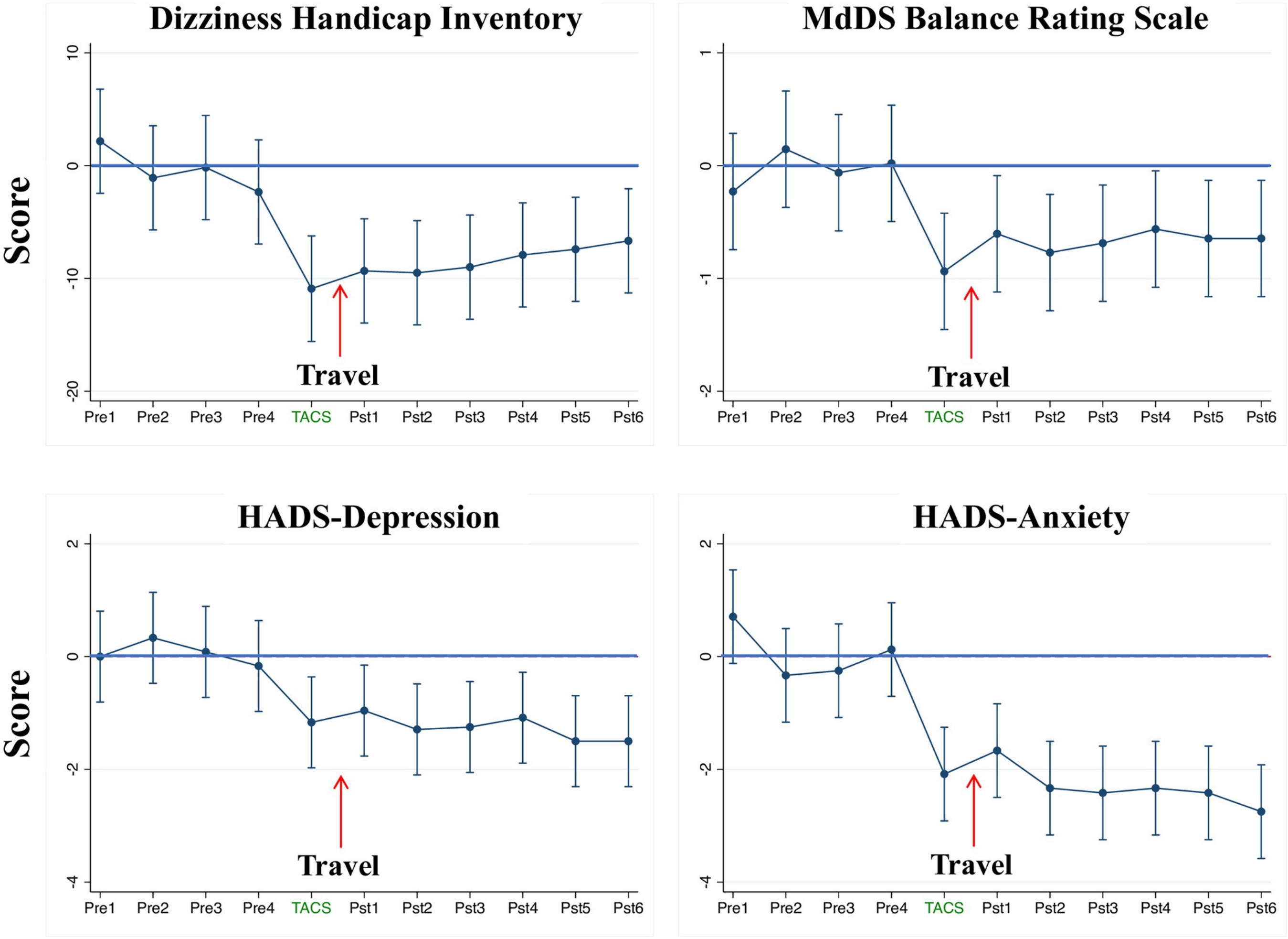

Fig 6. Longitudinal tACS effects on DHI, MBRS, and HAD scores. Linear prediction model of repeated measures ANOVA with 95% confidence intervals presented for four baseline measurements, post TMS week, and six weeks post treatment for the DHI, MBRS, and the HADS Anxiety and Depression components. Pre = pre TMS scores, Pst = post TMS scores.

https://doi.org/10.1371/journal.pone.0263558.g006

The comparable long-term efficacy of tACS to cTBS would suggest that tACS could be a less expensive, portable, and more accessible treatment method that may be available over a longer period of time than transcranial magnetic stimulation using either standard methods or with high frequency methods such as theta burst stimulation. Optimization of treatment parameters remains a formidable challenge, however as there are many factors that may influence both short-term and long-term durability of the treatment. These factors include the IAF, which brain region's IAF to target, treatment focality, number of stimulation sessions, latent conditions of stimulation, and concurrent medications. Optimal treatment dosing and timing is unknown. In the setting of this clinical trial, we used a fairly aggressive treatment strategy of stacking tACS sessions to avoid wearing off or rebound effects. However, real-time functional connectivity feedback is needed to know when to dose the next treatment session for sustained effects without overdosing.

Since motion triggers abound in the environment, there is likely not a permanent cure for MdDS. Re-exposure to prolonged passive motion can re-trigger MdDS symptoms or interfere with the consolidation phase of treatment. However, more effective methods for symptom mitigation and access to earlier treatment can be developed. There should be recognition that

one therapy cannot address all symptoms nor be optimized for all individuals. As in the study of cTBS over the occipital cortex or cerebellum in which each site worked better for different individuals, the anti-phase alpha vs in-phase alpha stimulation worked better for different individuals in this study.

We are aware that in some patient presentations in the clinical setting, there may be symptom overlap between MdDS, Persistent Postural Perceptual Dizziness (PPPD), vestibular migraine, and motion sickness [28–30]. In order to be very clear about the diagnosis of MdDS, we enrolled fewer than 30% of the potential participants that we screened in order to obtain a homogeneous group of individuals with MdDS. The individuals in the current study had clear-cut diagnoses of MdDS in which their symptoms were triggered by motion exposure and were temporarily relieved with re-exposure to motion. An unclear trigger for their symptoms was an exclusionary criterion for the study. Symptoms that are worsened by motion rather than relieved by motion are more typical for PPPD, vestibular migraine, and motion sickness. Although a history of migraine was not an exclusionary criterion for entry into the study, the individuals in our studies are advised in the consenting process that brain stimulation can potentially worsen headache. Individuals with bothersome headaches generally decline enrollment or wait until their headaches are better controlled before participating.

Limitations

A universal limitation in working with individuals with rare disorders, particularly one in which travel itself worsens the participants' symptoms, is sample size. MdDS is much less common than vestibular migraine or Meniere's disease but remains a relevant clinical disorder because of the intractability of symptoms and lack of effective treatment options [6,31–33]. In order to increase statistical power, we used an 'n-of-1' design and determined symptom change relative to the patient's own baseline [34]. The design itself included a control condition and two real stimulation conditions that would theoretically produce opposite physiological effects. Though in two cases, the participants chose the control condition, neither of those individuals nor the additional two participants who were allocated to the control condition noted any significant reduction in symptoms with repeated stimulation. We felt that it was not ethically allowable to give the participants a known true sham condition since a prior study showed that MdDS symptoms were exacerbated after sham rTMS treatment if participants with MdDS travelled home after the intervention [23]. The ethical position in this study was to allow the participants to choose the treatment for themselves with the possibility that they could choose the control condition. We note that the control condition was still active stimulation, simply not a protocol that we expected to drive the functional connectivity effects that we hypothesized were important.

Finally, a post-study elevation in symptoms after travel home is a persistent issue with neuromodulation studies on MdDS. We do advise our participants to take certain travel precautions prior to and during travel. However, the accumulation of study and travel-related fatigue as well as the exposure to motion during travel can lead to exacerbation of symptoms once the participant travels home. We did not see a significant worsening of symptoms at the first post-treatment week on a group level, but this could be an issue on an individual level. Due to participant fatigue, we limited data acquisition to 6-weeks post stimulation because any beneficial effect from the treatment should be observable within that time-period and longer observations could be contaminated by additional motion triggers.

Conclusions

This study explored the potential for fronto-occipital tACS to modulate the oscillating vertigo of MdDS showing that alpha frequency stimulation is preferable to gamma frequency

stimulation and that anti-phase stimulation is generally preferable to in-phase stimulation. In any individual, one may be better than the other, however. The IAF is likely an important factor in determining treatment parameters and should be explored further. Participants who were able to choose a preferred treatment protocol on a test day had higher treatment responses than those who noted no immediate effect. This suggests that a randomized n-of-1 test session of different protocols may be predictive of responses to multiple treatments. Since MdDS symptoms are chronic, are worsened by environmental stimuli, and can be re-triggered by subsequent motion, longer-term treatment is likely required for additive and sustained effects. Therefore, portable treatments such as tACS are worthy of further investigation.

Supporting information

S1 Checklist.
(PDF)

S1 Fig. Protocol selection by each participant based on best single session response.
(TIF)

S2 Fig. Comparison of three non-invasive treatment protocols for MdDS. A. Transcranial magnetic stimulation (TMS) at 1Hz and 10Hz over dorsolateral prefrontal cortex (DLPFC), B. Continuous theta burst stimulation over occipital cortex and cerebellar vermis, C. Transcranial alternating current stimulation (tACS) over fronto-occipital cortex.
(TIF)

S3 Fig. Comparison of post-stimulation effects up to 6-weeks after cTBS over occipital cortex/cerebellum versus fronto-occipital tACS. Mean score changes from baseline and 95% confidence interval for A. Dizziness Handicap Inventory, B. MdDS Balance Rating Scale, C. Hospital Anxiety and Depression Scale-Depression subscore, and D. Hospital Anxiety and Depression Scale-Anxiety subscore.
(TIF)

S1 File.
(DOCX)

Acknowledgments

The authors thank the many individuals who traveled long distances to participate in this study.

Author Contributions

Conceptualization: Yoon-Hee Cha.

Data curation: Yoon-Hee Cha, Diamond Gleghorn, Benjamin Chipper Doudican.

Formal analysis: Yoon-Hee Cha, Diamond Gleghorn, Benjamin Chipper Doudican.

Funding acquisition: Yoon-Hee Cha.

Investigation: Yoon-Hee Cha, Diamond Gleghorn, Benjamin Chipper Doudican.

Methodology: Yoon-Hee Cha.

Project administration: Yoon-Hee Cha, Diamond Gleghorn, Benjamin Chipper Doudican.

Resources: Yoon-Hee Cha.

Software: Yoon-Hee Cha.

Supervision: Yoon-Hee Cha.

Validation: Yoon-Hee Cha.

Visualization: Yoon-Hee Cha, Diamond Gleghorn, Benjamin Chipper Doudican.

Writing – original draft: Yoon-Hee Cha.

Writing – review & editing: Yoon-Hee Cha, Diamond Gleghorn, Benjamin Chipper Doudican.

References

1. Cha YH. Mal de debarquement. Semin Neurol. 2009; 29(5):520–7. Epub 2009/10/17. https://doi.org/10.1055/s-0029-1241038 PMID: 19834863; PubMed Central PMCID: PMC2846419.
2. Brown JJ, Baloh RW. Persistent mal de debarquement syndrome: a motion-induced subjective disorder of balance. Am J Otolaryngol. 1987; 8(4):219–22. Epub 1987/07/01. https://doi.org/10.1016/s0196-0709(87)80007-8 PMID: 3631419.
3. Cha YH, Brodsky J, Ishiyama G, Sabatti C, Baloh RW. Clinical features and associated syndromes of mal de debarquement. J Neurol. 2008; 255(7):1038–44. Epub 2008/05/27. https://doi.org/10.1007/s00415-008-0837-3 PMID: 18500497; PubMed Central PMCID: PMC2820362.
4. Hain TC, Hanna PA, Rheinberger MA. Mal de debarquement. Arch Otolaryngol Head Neck Surg. 1999; 125(6):615–20. Epub 1999/06/15. https://doi.org/10.1001/archotol.125.6.615 PMID: 10367916.
5. Cha YH, Cui Y. Rocking dizziness and headache: a two-way street. Cephalalgia. 2013; 33(14):1160–9. Epub 2013/05/16. https://doi.org/10.1177/0333102413487999 PMID: 23674832; PubMed Central PMCID: PMC5638651.
6. Cha YH, Cui YY, Baloh RW. Comprehensive Clinical Profile of Mal De Debarquement Syndrome. Front Neurol. 2018; 9:261. Epub 2018/06/06. https://doi.org/10.3389/fneur.2018.00261 PMID: 29867709; PubMed Central PMCID: PMC5950831.
7. Macke A, LePorte A, Clark BC. Social, societal, and economic burden of mal de debarquement syndrome. J Neurol. 2012; 259(7):1326–30. Epub 2012/01/11. https://doi.org/10.1007/s00415-011-6349-6 PMID: 22231864.
8. Arroll MA, Attree EA, Cha YH, Dancey CP. The relationship between symptom severity, stigma, illness intrusiveness and depression in Mal de Debarquement Syndrome. J Health Psychol. 2016; 21(7):1339–50. Epub 2014/10/22. https://doi.org/10.1177/1359105314553046 PMID: 25331814.
9. Cha YH, Shou G, Gleghorn D, Doudican BC, Yuan H, Ding L. Electrophysiological Signatures of Intrinsic Functional Connectivity Related to rTMS Treatment for Mal de Debarquement Syndrome. Brain Topogr. 2018; 31(6):1047–58. Epub 2018/08/14. https://doi.org/10.1007/s10548-018-0671-6 PMID: 30099627; PubMed Central PMCID: PMC6182441.
10. Chen Y, Cha YH, Li C, Shou G, Gleghorn D, Ding L, et al. Multimodal Imaging of Repetitive Transcranial Magnetic Stimulation Effect on Brain Network: A Combined Electroencephalogram and Functional Magnetic Resonance Imaging Study. Brain Connect. 2019; 9(4):311–21. Epub 2019/02/26. https://doi.org/10.1089/brain.2018.0647 PMID: 30803271; PubMed Central PMCID: PMC6533792.
11. Cha YH, Chakrapani S, Craig A, Baloh RW. Metabolic and functional connectivity changes in mal de debarquement syndrome. PLoS One. 2012; 7(11):e49560. Epub 2012/12/05. https://doi.org/10.1371/journal.pone.0049560 PMID: 23209584; PubMed Central PMCID: PMC3510214.
12. Ding L, Shou G, Yuan H, Urbano D, Cha YH. Lasting modulation effects of rTMS on neural activity and connectivity as revealed by resting-state EEG. IEEE Trans Biomed Eng. 2014; 61(7):2070–80. Epub 2014/04/02. https://doi.org/10.1109/TBME.2014.2313575 PMID: 24686227; PubMed Central PMCID: PMC5638649.
13. Yuan H, Shou G, Gleghorn D, Ding L, Cha YH. Resting State Functional Connectivity Signature of Treatment Effects of Repetitive Transcranial Magnetic Stimulation in Mal de Debarquement Syndrome. Brain Connect. 2017; 7(9):617–26. Epub 2017/10/03. https://doi.org/10.1089/brain.2017.0514 PMID: 28967282; PubMed Central PMCID: PMC5695731.
14. Smit CM, Wright MJ, Hansell NK, Geffen GM, Martin NG. Genetic variation of individual alpha frequency (IAF) and alpha power in a large adolescent twin sample. Int J Psychophysiol. 2006; 61(2):235–43. Epub 2005/12/13. https://doi.org/10.1016/j.ijpsycho.2005.10.004 PMID: 16338015.
15. Grandy TH, Werkle-Bergner M, Chicherio C, Schmiedek F, Lovden M, Lindenberger U. Peak individual alpha frequency qualifies as a stable neurophysiological trait marker in healthy younger and older

adults. Psychophysiology. 2013; 50(6):570–82. Epub 2013/04/05. https://doi.org/10.1111/psyp.12043 PMID: 23551082.

16. Haegens S, Cousijn H, Wallis G, Harrison PJ, Nobre AC. Inter- and intra-individual variability in alpha peak frequency. Neuroimage. 2014; 92:46–55. Epub 2014/02/11. https://doi.org/10.1016/j.neuroimage.2014.01.049 PMID: 24508648; PubMed Central PMCID: PMC4013551.

17. Grandy TH, Werkle-Bergner M, Chicherio C, Lovden M, Schmiedek F, Lindenberger U. Individual alpha peak frequency is related to latent factors of general cognitive abilities. Neuroimage. 2013; 79:10–8. Epub 2013/04/30. https://doi.org/10.1016/j.neuroimage.2013.04.059 PMID: 23624490.

18. Bland NS, Sale MV. Current challenges: the ups and downs of tACS. Exp Brain Res. 2019; 237 (12):3071–88. Epub 2019/10/18. https://doi.org/10.1007/s00221-019-05666-0 PMID: 31620829.

19. Tavakoli AV, Yun K. Transcranial Alternating Current Stimulation (tACS) Mechanisms and Protocols. Front Cell Neurosci. 2017; 11:214. Epub 2017/09/21. https://doi.org/10.3389/fncel.2017.00214 PMID: 28928634; PubMed Central PMCID: PMC5591642.

20. Antal A, Paulus W. Transcranial alternating current stimulation (tACS). Front Hum Neurosci. 2013; 7:317. Epub 2013/07/05. https://doi.org/10.3389/fnhum.2013.00317 PMID: 23825454; PubMed Central PMCID: PMC3695369.

21. Ahn S, Gleghorn D, Doudican B, Frohlich F, Cha YH. Transcranial Alternating Current Stimulation Reduces Network Hypersynchrony and Persistent Vertigo. Neuromodulation. 2021. Epub 2021/03/24. https://doi.org/10.1111/ner.13389 PMID: 33757158.

22. Cha YH, Gleghorn D, Doudican B. Occipital and Cerebellar Theta Burst Stimulation for Mal De Debarquement Syndrome. Otol Neurotol. 2019; 40(9):e928–e37. Epub 2019/08/23. https://doi.org/10.1097/MAO.0000000000002341 PMID: 31436631; PubMed Central PMCID: PMC6744334.

23. Cha YH, Deblieck C, Wu AD. Double-Blind Sham-Controlled Crossover Trial of Repetitive Transcranial Magnetic Stimulation for Mal de Debarquement Syndrome. Otol Neurotol. 2016; 37(6):805–12. Epub 2016/05/14. https://doi.org/10.1097/MAO.0000000000001045 PMID: 27176615; PubMed Central PMCID: PMC4907861.

24. Patterson JA, Amick RZ, Thummar T, Rogers ME. Validation of measures from the smartphone sway balance application: a pilot study. Int J Sports Phys Ther. 2014; 9(2):135–9. Epub 2014/05/03. PMID: 24790774; PubMed Central PMCID: PMC4004118.

25. Cha YH, Urbano D, Pariseau N. Randomized Single Blind Sham Controlled Trial of Adjunctive Home-Based tDCS after rTMS for Mal De Debarquement Syndrome: Safety, Efficacy, and Participant Satisfaction Assessment. Brain Stimul. 2016; 9(4):537–44. Epub 2016/04/28. https://doi.org/10.1016/j.brs.2016.03.016 PMID: 27117283.

26. Jacobson GP, Newman CW. The development of the Dizziness Handicap Inventory. Arch Otolaryngol Head Neck Surg. 1990; 116(4):424–7. Epub 1990/04/01. https://doi.org/10.1001/archotol.1990.01870040046011 PMID: 2317323.

27. Zigmond AS, Snaith RP. The hospital anxiety and depression scale. Acta Psychiatr Scand. 1983; 67 (6):361–70. Epub 1983/06/01. https://doi.org/10.1111/j.1600-0447.1983.tb09716.x PMID: 6880820.

28. Staab JP, Eckhardt-Henn A, Horii A, Jacob R, Strupp M, Brandt T, et al. Diagnostic criteria for persistent postural-perceptual dizziness (PPPD): Consensus document of the committee for the Classification of Vestibular Disorders of the Barany Society. J Vestib Res. 2017; 27(4):191–208. Epub 2017/10/19. https://doi.org/10.3233/VES-170622 PMID: 29036855.

29. Lempert T, Olesen J, Furman J, Waterston J, Seemungal B, Carey J, et al. Vestibular migraine: diagnostic criteria. J Vestib Res. 2012; 22(4):167–72. Epub 2012/11/13. https://doi.org/10.3233/VES-2012-0453 PMID: 23142830.

30. Golding JF. Motion sickness. Handb Clin Neurol. 2016; 137:371–90. Epub 2016/09/18. https://doi.org/10.1016/B978-0-444-63437-5.00027-3 PMID: 27638085.

31. Cha YH. Less common neuro-otologic disorders. Continuum (Minneap Minn). 2012; 18(5 Neuro-otology):1142–57. Epub 2012/10/09. https://doi.org/10.1212/01.CON.0000421623.56525.11 PMID: 23042064.

32. Neuhauser HK, von Brevern M, Radtke A, al. e. Epidemiology of vestibular vertigo: a neurotologic survey of the general population. Neurology. 2005; 65(6):898–904. https://doi.org/10.1212/01.wnl.0000175987.59991.3d PMID: 16186531

33. Neuhauser HK, Radtke A, von Brevern M, Feldmann M, Lezius F, Ziese T, et al. Migrainous vertigo: prevalence and impact on quality of life. Neurology. 2006; 67(6):1028–33. Epub 2006/09/27. https://doi.org/10.1212/01.wnl.0000237539.09942.06 PMID: 17000973.

34. Vieira R, McDonald S, Araujo-Soares V, Sniehotta FF, Henderson R. Dynamic modelling of n-of-1 data: powerful and flexible data analytics applied to individualised studies. Health Psychol Rev. 2017; 11 (3):222–34. Epub 2017/06/21. https://doi.org/10.1080/17437199.2017.1343680 PMID: 28629262.
